# Nephronectin (NPNT) is a Crucial Determinant of Idiopathic Pulmonary Fibrosis: Modulating Cellular Senescence via the ITGA3/YAP1 Signaling Axis

**DOI:** 10.1002/advs.202501956

**Published:** 2025-05-30

**Authors:** Jiayu Guo, Yan Wang, Qiudi Liu, Zhaoyang Luo, Xiaomu Tian, Yuquan Wang, Yang Liu, Zhiwei Ning, Yingying Guo, Huiying Gao, Xinyue Wang, Jinglong Feng, Mengmeng Liu, Dina Saifullina, Yixin Zhang, Tengfei Pan, Yu Bian, Tao Ban, Tianyu Li, Yunyan Gu, Haihai Liang

**Affiliations:** ^1^ State Key Laboratory of Frigid Zone Cardiovascular Diseases (SKLFZCD) Department of Pharmacology (State Key Laboratory–Province Key Laboratories of Biomedicine–Pharmaceutics of China Key Laboratory of Cardiovascular Research Ministry of Education) College of Pharmacy Harbin Medical University Harbin 150081 China; ^2^ Department of Systems Biology College of Bioinformatics Science and Technology Harbin Medical University Harbin 150081 China; ^3^ Research Unit of Noninfectious Chronic Diseases in Frigid Zone (2019RU070) Chinese Academy of Medical Sciences Harbin 150081 China

**Keywords:** cellular senescence, hippo, integrin α3, nephronectin, pulmonary fibrosis

## Abstract

Idiopathic pulmonary fibrosis (IPF) is a prototype of chronic, progressive, and fibrotic lung disease. While advancing age is recognized as the most significant risk factor for both the development and mortality associated with pulmonary fibrosis, precise mechanisms underlying this association remain elusive. Here, Nephronectin (NPNT) is identified as an antiaging molecule, a potential major regulator of the progression of pulmonary fibrosis. In IPF patients, a marked reduction in NPNT expression is detected in lung tissues, which correlated with a decline in lung function. The study reveals that NPNT deficiency exacerbates bleomycin‐induced senescence in alveolar epithelial cells, potentially intensifying fibrosis severity due to diminishes extracellular matrix turnover. Conversely, NPNT overexpression in the alveolar epithelium improves lung respiratory function and enhances resistance to aging and fibrosis. Mechanistically, NPNT inhibits the hyperactivation of LATS1 and MOB1, facilitates YAP1 nuclear translocation, and suppresses YAP1 ubiquitination and degradation, contingent upon the interaction between NPNT and ITGA3. Notably, pharmacological elevation of NPNT protein levels using Escin has been shown to alleviate pulmonary fibrosis and improve lung function in mice. The findings shed light on the key mechanism underlying stress‐induced senescence and fibrosis, and offer a promising framework for interventions targeting aging‐related diseases.

## Graphical abstract

Through a comprehensive multi‐omics analysis, this study identifies a marked reduction in Nephronectin (NPNT) expression within fibrotic lung tissue. This reduction impairs the binding capability to the ITGA3 receptor, consequently causing YAP1 to persist in the cytoplasm, where it undergoes degradation. The sequence of events contributes to the senescence of alveolar epithelial cells and facilitates the progression of pulmonary fibrosis.

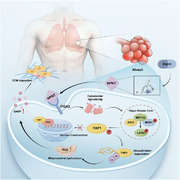

## 1 Introduction

Idiopathic pulmonary fibrosis (IPF) is an age‐related disorder characterized by damage to the alveolar epithelium, leading to progressive scarring and ultimately respiratory failure and death.^[^ ^1^ ^]^ In this disease, impaired epithelial regeneration triggers fibroblast activation, which results in excessive matrix deposition and contraction, increasing lung stiffness and hindering gas exchange.^[^ ^2^ ^]^ Nintedanib and Pirfenidone are currently recommended drugs for IPF in clinical practice guidelines across multiple countries, as they have been shown to slow the decline in lung function in IPF patients.^[^ ^3^, ^4^ ^]^ However, there is a lack of robust clinical trial data to confirm the long‐term survival benefits of antifibrotic therapies. Despite the significance of fibrosis in wound healing, there remains an unmet medical need to identify effective antifibrosis treatments.

Senescent cells are prevalent in tissues affected by pulmonary fibrosis and play a significant role in the pathogenesis of associated diseases.^[^ ^5^ ^]^ Cellular senescence is a state of irreversible cell cycle arrest characterized by the upregulation of cell cycle inhibitors, particularly CDKN1A (P21) and CDKN2A (P16), elevated levels of reactive oxygen species (ROS), as well as the secretion of inflammatory cytokines, chemokines, and matrix remodeling enzymes, globally known as senescence‐associated secretory phenotype (SASP).^[^ ^6^ ^]^ The removal of senescent cells has been shown to improve the phenotype of bleomycin(BLM)‐induced pulmonary fibrosis.^[^ ^7^ ^]^ Consequently, there is an urgent need to explore the precise molecular mechanism underlying lung cell aging and damage repair during the progression of IPF, paving the way for developing novel treatment techniques.

The extracellular matrix (ECM) is a vital noncellular component of tissues that provides a dynamic living environment for various cell types and significantly influences their biological activities.^[^ ^8^ ^]^ Recent studies underscore the important role of ECM in tissue repair. Versican has been shown to promote cardiomyocyte proliferation and cardiac repair.^[^ ^9^ ^]^ Additionally, Darrell Pilling et al. revealed that Slit2, secreted by fibroblasts, might suppress fibrocyte differentiation and fibrosis.^[^ ^10^ ^]^ The whole genome sequencing of lung tissue samples has revealed that the enrichment of lung ECM receptor interaction pathways is the most significant change in gene expression with age, highlighting the role of ECM in both lung health and disease.^[^ ^11^ ^]^ Nephronectin (NPNT) is a highly conserved ECM protein that serves as a major ligand for the integrin α8β1. Advancements in sequencing technology have facilitated a more in‐depth exploration of the role of NPNT in pulmonary biology. A meta‐analysis has revealed that the FEV(1)‐related locus (INTS12‐GSTCD‐NPNT) is associated with genome‐wide significance, underscoring its potential role in pulmonary function.^[^ ^12^ ^]^ NPNT is also crucial for maintaining right lobe separation during embryonic development, and its absence in the alveolar basement membrane can impede or delay the resolution of inflammation and injury.^[^ ^13^, ^14^ ^]^ In the present study, we sought to elucidate the effect of NPNT on the development of pulmonary fibrosis.

In this study, we found that NPNT was decreased in the lung tissues of IPF patients and in a mouse model of pulmonary fibrosis. During lung injury, NPNT was downregulated in alveolar epithelial type II (AT2) cells, and its interaction with integrin alpha‐3 (ITGA3) was compromised. This disruption led to the senescence of alveolar epithelial cells, the release of a substantial SASP, and facilitated ECM deposition and fibroblast differentiation. These findings suggest that the activation of NPNT signaling may constitute a novel and efficacious therapeutic strategy for the treatment of pulmonary fibrosis.

## 2 Result

### 2.1 Decreased NPNT Expression in the Lungs of IPF Patients and Mice with Experimental Pulmonary Fibrosis

**Figure 1:**
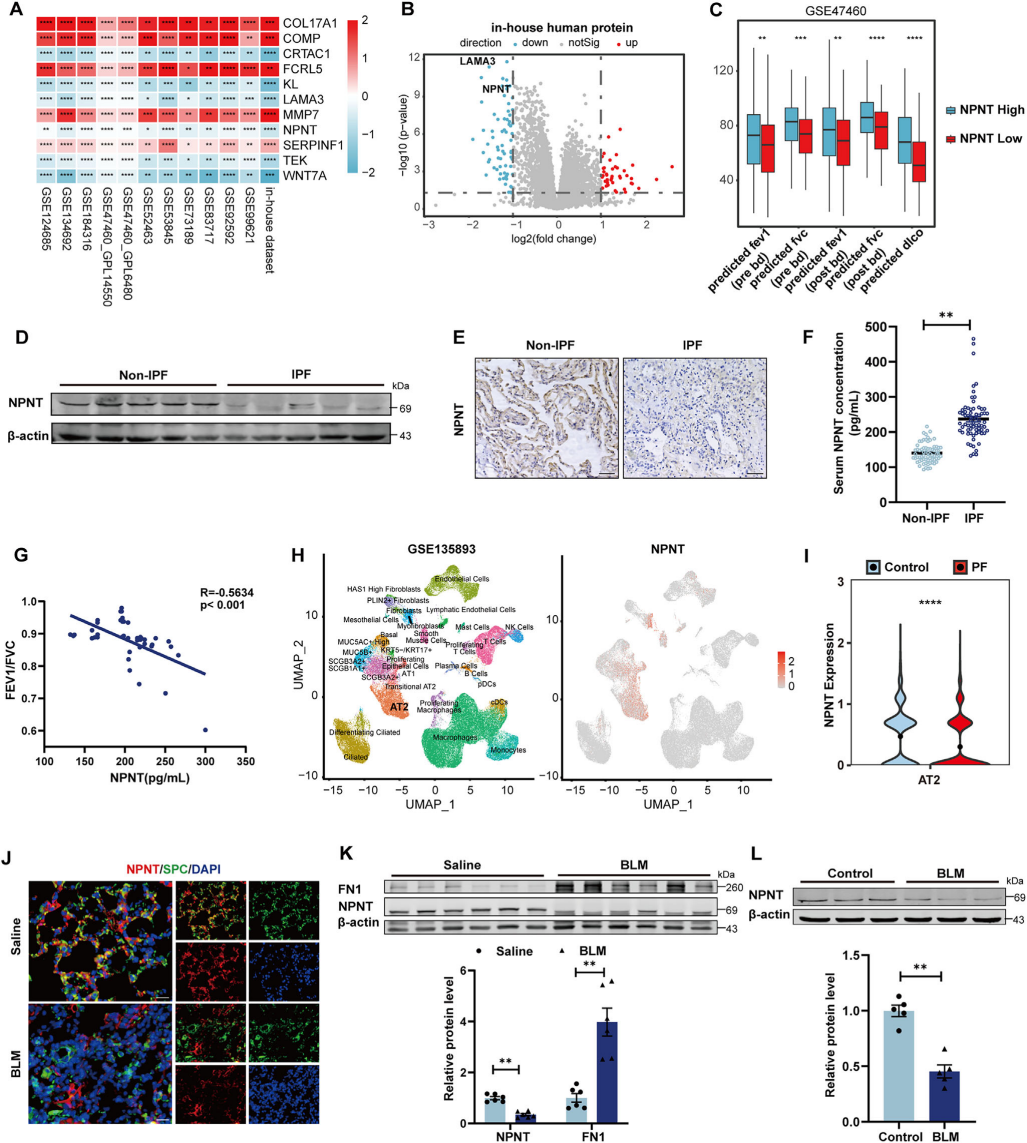
The expression of NPNT was downregulated in fibrotic lung tissues and injured alveolar epithelial cells. A) Transcriptome RNA‐seq datasets illustrating the transcriptional expression of secreted protein genes in IPF patients compared to the control group. Colors from red to blue represent log2 fold change values, with the IPF group relative to the control group. Statistical significance determined by two‐sided Wilcoxon rank‐sum test: ^*^ *p* < 0.05, ^**^ *p* < 0.01, ^***^ *p* < 0.001, ^****^ *p* < 0.0001. B) Volcano plot based on data analysis of proteomics in IPF patients. *n* = 9 per group. C) According to the mean value of NPNT expression value, all samples were divided into NPNT high expression group and NPNT low expression group, and the corresponding lung function data (GSE47460) of patients were analyzed. D,E) The protein expression and tissue localization of NPNT were detected by immunoblotting and immunohistochemistry (*n* = 5 per group). F) Elisa assay was used to detect NPNT protein concentrations in the serum of healthy volunteers and IPF patients (*n* = 69 per group). G) Pearson correlation analysis of serum NPNT concentration and FEV1/FVC in patients with lung fibrosis (*n* = 41). H) The expression of NPNT in single‐cell transcriptome data derived from human PF samples has been analyzed, utilizing dataset author annotations from all samples within GSE135893. I) Comparison of NPNT expression in AT2 cells between the control group and PF group in the GSE135893 dataset, evaluated using a two‐sided Wilcoxon rank‐sum test (^****^ *p* < 0.0001). J) Tissue immunofluorescence staining identified the expression of NPNT in AT2 cells (*n* = 4). Scale bars = 20 µm. K) Western blotting and quantification showed the protein expression of fibronectin 1 (FN1) and NPNT in lung tissue samples of Saline‐ and BLM‐treated mice. (*n* = 6 samples per group). (L) The protein level of NPNT in MLE‐12 cells after BLM‐induced injury. (*n =* 5 samples per group). Data are presented as mean±SEM. ^*^ *p* < 0.05, ^**^ *p* < 0.01, ^***^ *p* < 0.001, ^****^ *p* < 0.0001.

To investigate the role of ECM proteins in pulmonary fibrosis, we analyzed 12 sets of transcriptome data and identified significant differential expression of 11 ECM‐related genes (**Figure** **1**A). Utilizing these identified genes, integrated proteomic analysis of IPF patients and bleomycin (BLM)‐induced pulmonary fibrosis in mice, we discovered that NPNT and lamininα3 (LAMA3) were the only genes consistently downregulated at both mRNA and protein levels in fibrotic lung tissues (Figure 1A,B; Figure S1A, Supporting Information). While lung‐specific loss of LAMA3 aggravated BLM‐induced pulmonary fibrosis, our attention was drawn to NPNT due to its high enrichment in the lungs compared to other organs (Figure S1B,C, Supporting Information). We investigated its regulatory effect on pulmonary fibrosis and found that patients with higher NPNT expression tended to have better lung function, as measured by forced expiratory volume in the first second (FEV1), forced vital capacity (FVC), and diffusion lung capacity for carbon monoxide (DLCO) (Figure 1C). Notably, the mRNA and protein levels of NPNT were significantly reduced in the lungs of IPF patients (Figure 1D,E; Figure S1D, Supporting Information), yet its expression was upregulated in serum (Figure 1F). This phenomenon may be ascribed to two potential mechanisms: the compromised binding of NPNT to its receptors, resulting in a subsequent loss of ligand functionality under conditions of pulmonary injury; or the release of NPNT into the circulatory system due to cellular death or damage, thereby causing elevated serum NPNT levels. The serum protein expression of NPNT negatively correlated with the FEV1/FVC ratio, a measure of lung function (Figure 1G).

Single‐cell data from pulmonary fibrosis (PF) patients, analyzed through clustering, showed that NPNT was predominantly enriched in alveolar epithelial cells, followed by Club cells and myofibroblasts (Figure 1H; Figure S1E, Supporting Information). In fibrotic lung tissues, NPNT expression was markedly diminished in alveolar epithelial type I (AT1) cells, AT2 cells, and SCGB3A2‐positive Club cells, with no notable change in myofibroblasts (Figure 1I; Figure S1F, Supporting Information). AT2 cells are of particular interest as they serve as the principal stem cells of the alveolar epithelium, possessing the ability to self‐proliferate and differentiate into AT1 cells in response to lung injury.^[^ ^15^ ^]^ Immunofluorescence analysis showed that NPNT expression was abundant in AT2 cells in the lung tissue of mice in the Saline group, with a decrease in fluorescence intensity and a reduction in the number of NPNT^+^/SPC^+^ cells after BLM‐induced lung injury (Figure 1J). Consistently, the expression levels of NPNT were significantly decreased in BLM‐induced mouse lung tissues and in the mouse alveolar epithelial cell line MLE‐12 (Figure 1K,L; Figure S1G, Supporting Information). In summary, these findings collectively demonstrate that NPNT expression is significantly reduced in the lung tissues of IPF patients, with a marked loss of expression specifically in AT2 cells. This observation suggests a potential association between NPNT deficiency and the development of pulmonary fibrosis.

### 2.2 NPNT Deficiency Exacerbates BLM‐Induced Pulmonary Fibrosis

**Figure 2:**
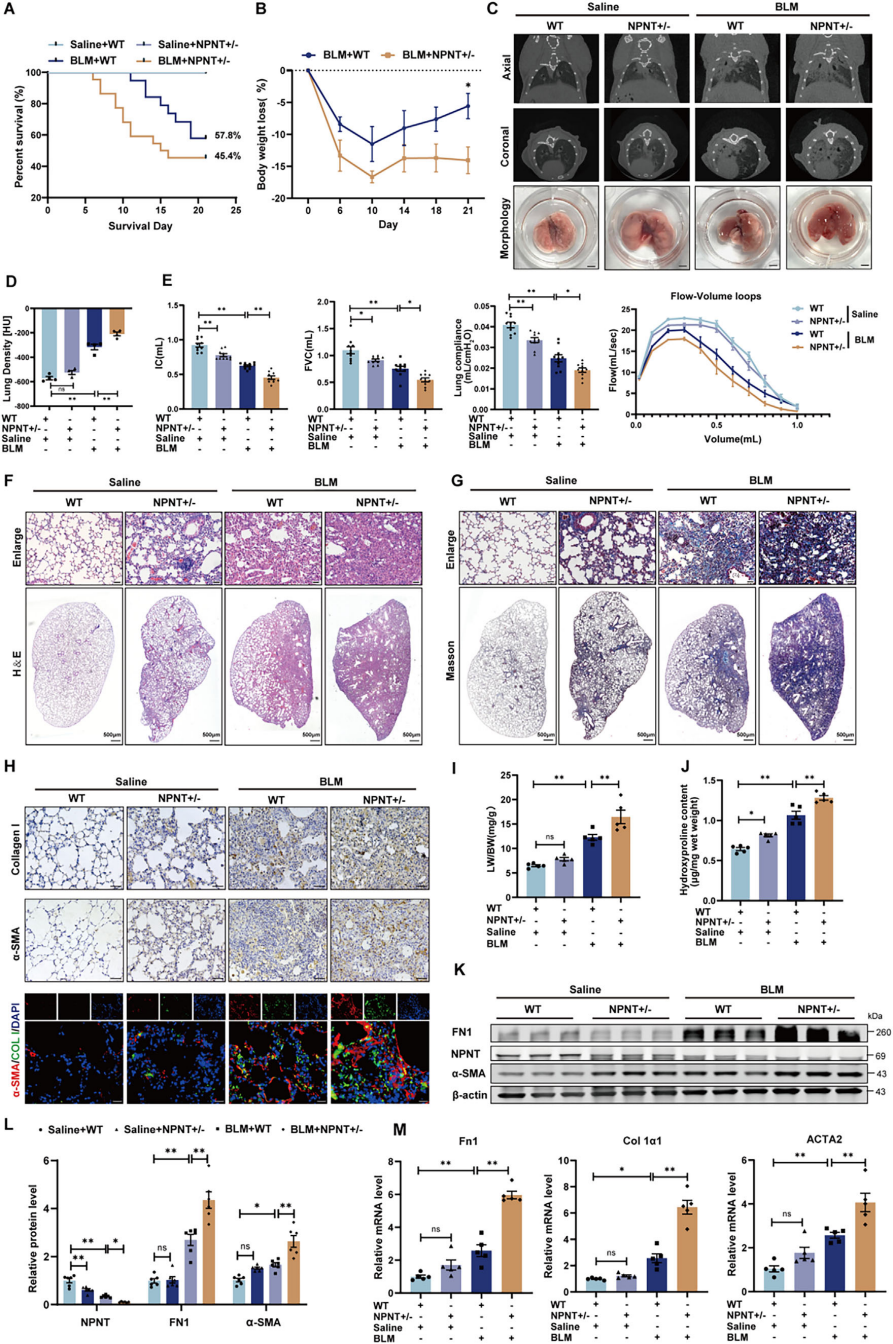
NPNT deficiency exacerbated BLM‐induced pulmonary fibrosis and pulmonary dysfunction in mice. A) Kaplan‐Meier analysis of WT and NPNT+/− mice 3 weeks after Saline or BLM surgery. Saline+WT n = 10, Saline+NPNT+/− *n* = 10, BLM+WT *n* = 19, BLM+NPNT+/− *n* = 22. B) Percentage of weight loss in mice over time after BLM induction. C) Micro‐CT imaging showed the shadow area of the mouse lung, and the bottom group was the morphology of mouse lung tissue. *n* = 4. Scale bars = 2 mm. D) Lung density in Hounsfield units (HU) based on micro‐CT images. E) After 3 weeks of Saline or BLM stimulation, the lung function of WT and NPNT+/− mice was tested, including IC, FVC, lung compliance, and flow‐volume loop (*n* = 8–10 per group). F,G) Representative images of H&E and Masson staining of WT and NPNT+/− mice 3 weeks after intratracheal injection of Saline or BLM. Scale bar, 50 µm. H) The pulmonary collagen deposition and myofibroblast formation of WT and NPNT+/− after BLM induction were detected by immunohistochemical staining (Scale bar, 50 µm) and immunofluorescence staining (Scale bar, 20 µm). *n* = 4 per group. I) The ratio of lung weight to body weight of mice was weighed and calculated (*n* = 5 per group). J) The content of hydroxyproline in mouse lung tissue was measured to reflect the decomposition of connective tissue (*n* = 5 per group). K,L) Western blotting and quantification showed protein levels of FN1, α‐SMA, and NPNT in WT and NPNT+/− mice after Saline or BLM injection. *n* = 6 per group. (M) Quantification of mRNA levels of Fn1, Col 1α1, and ACTA2. *n* = 5 samples per group. Data are presented as mean ± SEM. ^*^ *p* < 0.05, ^**^ *p* < 0.01.

To determine whether the absence of NPNT expression promoted fibrogenesis, we assessed the response of NPNT knockout mice to BLM‐induced pulmonary fibrosis. Given the lower birth rate of NPNT homozygous mutant mice, heterozygous NPNT mice (NPNT+/−) were utilized for subsequent investigation (Figure S2A–C, Supporting Information). After BLM induction, NPNT+/− mice exhibited a decline in survival rate from 57.8% to 45.4% and reduced body weight, contrasting with wild‐type (WT) mice (**Figure** **2**A,B). Micro‐CT imaging confirmed that NPNT+/− mice displayed enhanced pulmonary fibrosis and more severe lung tissue density following BLM exposure (Figure 2C,D). Lung function tests revealed that NPNT+/− mice were more vulnerable to BLM‐induced lung dysfunction, as evidenced by reduced inspiratory capacity (IC), FVC, lung compliance, and altered flow‐volume loops in NPNT+/− mice (Figure 2E), suggesting a significant association between NPNT expression and lung function in mice. H&E staining and Masson's trichrome staining revealed thicker alveolar walls and increased collagen deposition in NPNT+/− mice. After BLM induction, these mice displayed more severe inflammatory cell infiltration, alveolar collapse, and a broader distribution of collagen fibers (Figure 2F,G). Increased protein levels of Collagen I and accumulation of α‐SMA^+^ myofibroblasts further substantiated the more severe fibrosis in NPNT+/− mice (Figure 2H). As shown in Figure 2I, the lung‐to‐body weight ratio increased in NPNT+/− mice following lung injury. Hydroxyproline accumulation in lung tissue was also higher in NPNT+/− mice compared to WT mice (Figure 2J). Additionally, qRT‐PCR and Western blot analyses showed elevated mRNA and protein levels of fibrosis‐related markers in the lung tissues of NPNT+/− mice, indicating that NPNT knockdown promoted ECM deposition and fibroblast‐to‐myofibroblast differentiation (Figure 2K–M). These findings collectively suggest that the dysregulation of NPNT expression plays a contributory role in the progression of pulmonary fibrosis.

### 2.3 Conditional Overexpression of NPNT in AT2 Cells Improves Lung Function and Attenuates Pulmonary Fibrosis in Mice

**Figure 3:**
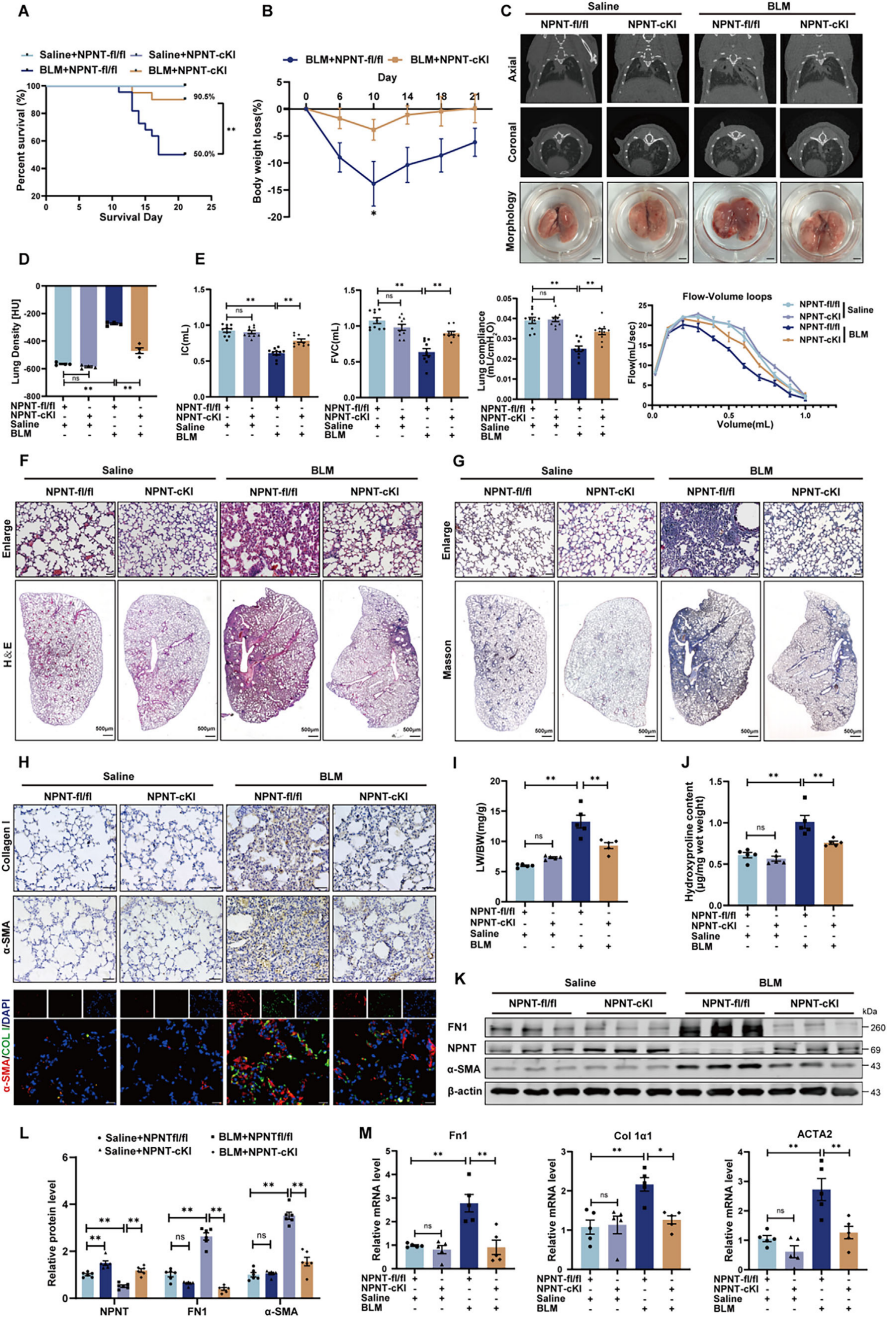
Specific overexpression of NPNT in AT2 cells inhibited the progression of pulmonary fibrosis and improved lung function in mice. A) Percentages of surviving NPNT‐cKI and NPNT‐fl/fl mice were plotted over a 21‐day period post‐BLM or ‐Saline administration. Saline+NPNT‐fl/fl *n* = 10, Saline+NPNT‐cKI *n* = 10, BLM+NPNT‐fl/fl *n = *22, BLM+NPNT‐cKI *n = *20. B) During the observation period of 21 days, the corresponding percentage of weight loss in each group was calculated. C) Representative images of the different groups were determined by micro‐CT imaging. Healthy lungs are black, and diseased lungs with elevated density are white. The appearance of lung tissue was shown in the bottom row (*n = *4 per group). Scale bars = 2 mm. D) Lung density in Hounsfield units (HU). *n = *4. E) Lung function parameters, including IC, FVC, lung compliance, and flow‐volume loop, among different groups were compared at day 21 post‐BLM or saline administration (*n = *8–10 per group). F,G) NPNT‐fl/fl and NPNT‐cKI mice were treated with H&E staining and Masson staining on day 21 after BLM or Saline administration. The image on the upper panel is magnified from the micrograph on the lower panel. H) Immunohistochemical staining (Scale bar, 50 µm) and immunofluorescence staining (Scale bar, 20 µm) showed the expression levels of Collagen I and α‐SMA in lung tissues of different groups. I) Ratio of lung weight (LW) to body weight (BW) (*n = *5 per group). J) Hydroxyproline content of lungs from NPNT‐fl/fl and NPNT‐cKI mice after BLM injection (*n = *5 per group). K,L) The inhibitory effect of overexpression of NPNT on FN1 and α‐SMA protein levels was detected by Western blot. *n = *6 per group. M) Quantitative real‐time PCR analysis of Fn1, Col 1α1, and ACTA2 mRNA levels in lung homogenates of BLM‐challenged NPNT‐fl/fl and NPNT‐cKI mice. *n = *5 samples per group. Data are presented as mean±SEM. ^*^ *p* < 0.05, ^**^ *p* < 0.01.

To investigate the potential therapeutic effects of NPNT overexpression in the alveolar epithelium on experimental pulmonary fibrosis, we generated mice with conditional overexpression of NPNT specifically in AT2 cells by crossing NPNT‐fl/fl mice with Sftpc‐Cre mice (Figure S2D–F, Supporting Information). Immunofluorescence staining confirmed increased NPNT expression in SPC^+^ cells (Figure S2G, Supporting Information). Following three weeks of BLM administration, mice with NPNT overexpression exhibited improved survival rate and regained weight (**Figure** **3**A,B). Micro‐CT scans demonstrated that NPNT overexpression effectively preserved lung structure and reduced pulmonary parenchymal lesions following BLM induction (Figure 3C,D). Enhanced NPNT expression also facilitated the recovery of lung function, as evidenced by improvement in IC, FVC, lung compliance, and flow‐volume loop (Figure 3E), as well as improved lung tissue structural lesions (Figure 3F). Masson's trichrome staining and immunohistochemistry further revealed that NPNT overexpression significantly diminished the extent of fibrosis and suppressed the excessive deposition of collagen after BLM treatment (Figure 3G,H). Similarly, NPNT overexpression reduced the presence of α‐SMA^+^ myofibroblasts in lung tissues (Figure 3H). After BLM stimulation, compared with NPNT‐fl/fl mice, NPNT‐cKI mice displayed a lower lung weight‐to‐body weight ratio and hydroxyproline content (Figure 3I,J). At mRNA and protein levels, NPNT overexpression significantly suppressed the expression of ECM components FN1 and Collagen I, as well as reduced α‐SMA (Figure 3K–M). These results indicate that NPNT overexpression may impede BLM‐induced ECM deposition and the degree of pulmonary fibrosis.

### 2.4 NPNT as a Modulator of Alveolar Epithelial Cell Senescence

**Figure 4:**
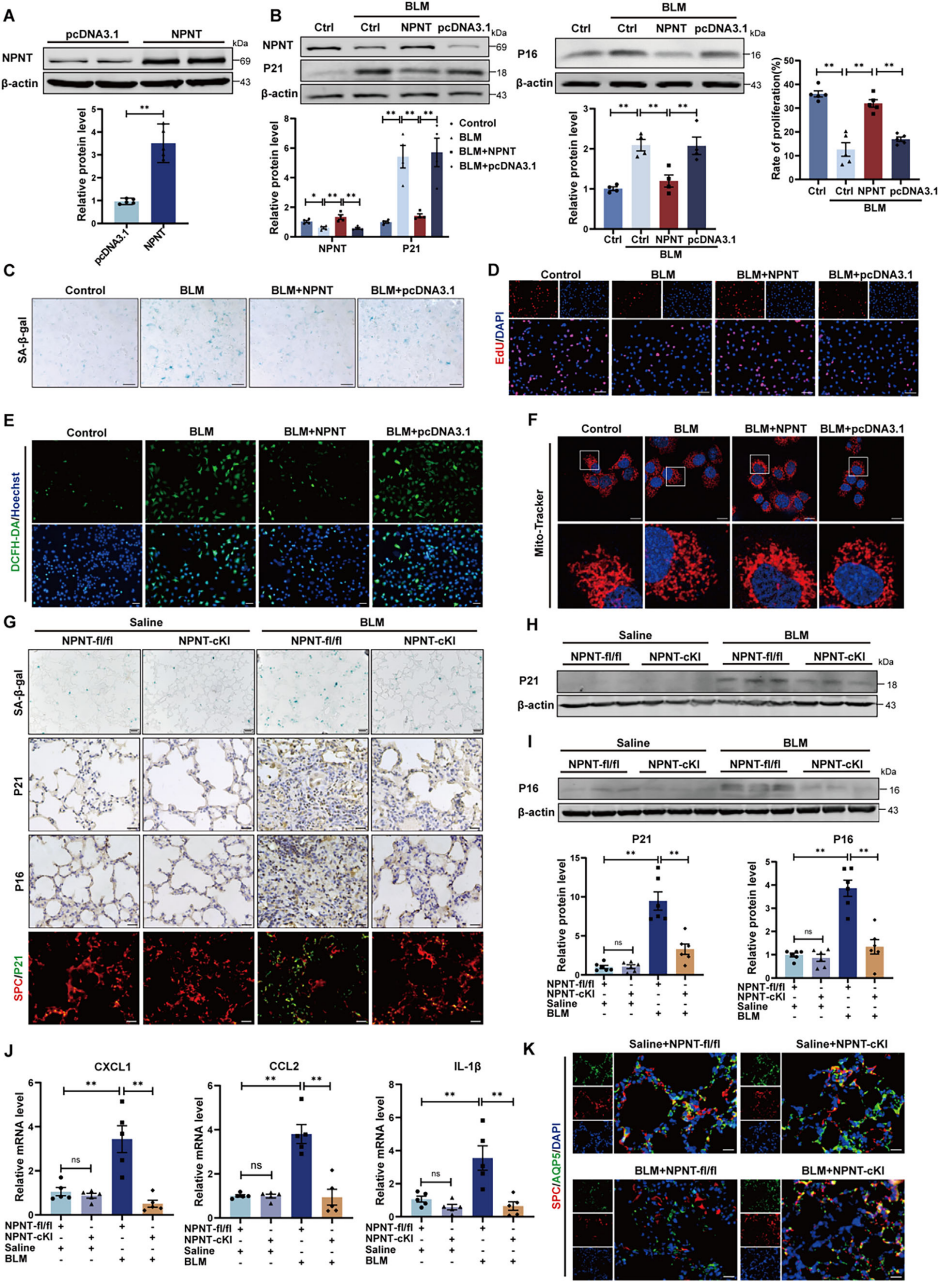
Overexpression of NPNT inhibited BLM‐induced epithelial cell senescence. A) Efficiency verification of NPNT overexpression plasmid (*n = *5 per group). B) Western blot analysis of P16 and P21 expression. *n = *4 samples per group. C) SA‐β‐gal staining was used to evaluate the senescence of MLE‐12 cells transfected with NPNT overexpression plasmid after BLM treatment. Scale bar, 50 µm. D) The proliferation ability of different groups of MLE‐12 cells was calculated by EdU‐positive cells. *n = *5. Scale bar, 50 µm. E) The ROS levels in cells overexpressing NPNT after BLM treatment were labeled with DCFH‐DA probe. *n = *4 samples per group. Scale bar, 50 µm. F) The morphological changes of mitochondria in living cells were shown by representative images of immunofluorescence. Scale bar, 15 µm. G) Representative images of SA‐β‐gal (Scale bar, 50 µm), immunohistochemical (Scale bar, 20 µm), and immunofluorescence staining (Scale bar, 20 µm) in NPNT‐fl/fl and NPNT‐cKI mice 3 weeks after Saline or BLM administration. *n = *4 samples per group. H,I) The protein levels of P21 and P16 were quantitatively analyzed by Western blot (*n = *6 per group). J) Determination of CXCL1, CCL2, and IL‐1β mRNA levels in lung tissues of NPNT‐fl/fl and NPNT‐cKI mice after Saline or BLM injection (*n = *5 per group). K) Immunofluorescence staining was used to detect the expression changes of SPC and AQP5 in mouse lung sections. Scale bar, 20 µm. Data are presented as mean±SEM. ^*^ *p* < 0.05, ^**^ *p* < 0.01.

Previous studies have shown that NPNT regulates skeletal muscle homeostasis and counteracts skeletal muscle aging in primates during physiological aging.^[^ ^16^ ^]^ Building upon this finding and considering the cellular localization of NPNT, we proposed the hypothesis that NPNT may inhibit pulmonary fibrosis by modulating alveolar epithelial cell senescence. We constructed an NPNT overexpression plasmid, and observed that an increased NPNT level in MLE‐12 cells significantly suppressed the protein expression of senescence‐associated markers P21 and P16, and reduced cellular β‐galactosidase accumulation (**Figure** **4**A–C). EdU fluorescence assays displayed that NPNT overexpression significantly attenuated the inhibitory effect of BLM on MLE‐12 cell proliferation (Figure 4D). Notably, cell senescence is often accompanied by mitochondrial dysfunction and oxidative damage.^[^ ^17^ ^]^ ROS staining indicated that NPNT could effectively suppress BLM‐induced ROS production and partially restore mitochondrial length and morphology (Figure 4E,F). Subsequently, we employed the human pulmonary alveolar epithelial cells type II (HPAEpiC‐II) for our investigations. We found that NPNT reduced β‐galactosidase accumulation and ROS production compared to the BLM‐treated group (Figure S3A,B, Supporting Information). NPNT overexpression decreased P21 protein level and partially restored cell proliferation affected by BLM (Figure S3C,D, Supporting Information). These findings suggest that NPNT plays a role in inhibiting BLM‐induced cellular senescence in HPAEpiC‐II cells, aligning with the results obtained in MLE‐12 cells. We thereafter confirmed the inhibitory effect of NPNT on the senescence phenotype in vivo. Senescence‐associated β‐galactosidase (SA‐β‐gal) staining revealed that the β‐galactosidase content of NPNT‐cKI mice post‐BLM treatment was lower than that in NPNT‐fl/fl mice (Figure 4G). NPNT overexpression significantly dampened the upregulation of P21 and P16 in the lungs. Immunofluorescence results showed a reduction in P21^+^/SPC^+^ cells in NPNT‐cKI mice following BLM treatment (Figure 4G). Western blot results confirmed that the protein levels of P21 and P16 were reduced in the lung tissues of NPNT‐overexpressing mice (Figure 4H,I). The production and secretion of SASP components are hallmarks of cellular senescence, and NPNT significantly suppressed the production of CXCL1, CCL2, and IL‐1β induced by BLM (Figure 4J). The number of AQP5/SPC labeled AT1/AT2 cells in NPNT‐cKI mice remarkedly increased following BLM administration (Figure 4K).

We also constructed siRNA to knockdown NPNT expression in MLE‐12 cells (Figure S4A, Supporting Information). Silencing NPNT promoted the protein levels of P21 and P16, and increased SA‐β‐gal activity, confirming the induction of senescence by siNPNT (Figure S4B,C, Supporting Information). In line with these observations, cells with silenced NPNT exhibited decreased proliferation capacity (Figure S4D, Supporting Information). Additionally, suppressing NPNT expression promoted ROS production and mitochondrial morphological changes in cells (Figure S4E,F, Supporting Information). Compared with WT mice, NPNT+/− mice were more susceptible to BLM‐induced lung aging, characterized by increased β‐galactosidase content and upregulated expression of senescence‐related markers, as well as increased P21 and SPC double‐positive cells (Figure S4G–I, Supporting Information). The absence of NPNT increased the levels of inflammatory factors and chemokines, impeding the regeneration of alveolar epithelial cells (Figure S4J,K, Supporting Information). Collectively, these results demonstrate that NPNT levels are inversely correlated with cellular senescence, and the lack of NPNT promotes cell senescence, whereas NPNT overexpression in AT2 cells aids in the recovery and regeneration of damaged alveolar epithelial cells.

### 2.5 NPNT Combats Aging by Facilitating YAP1 Nuclear Translocation and Preventing its Degradation

**Figure 5:**
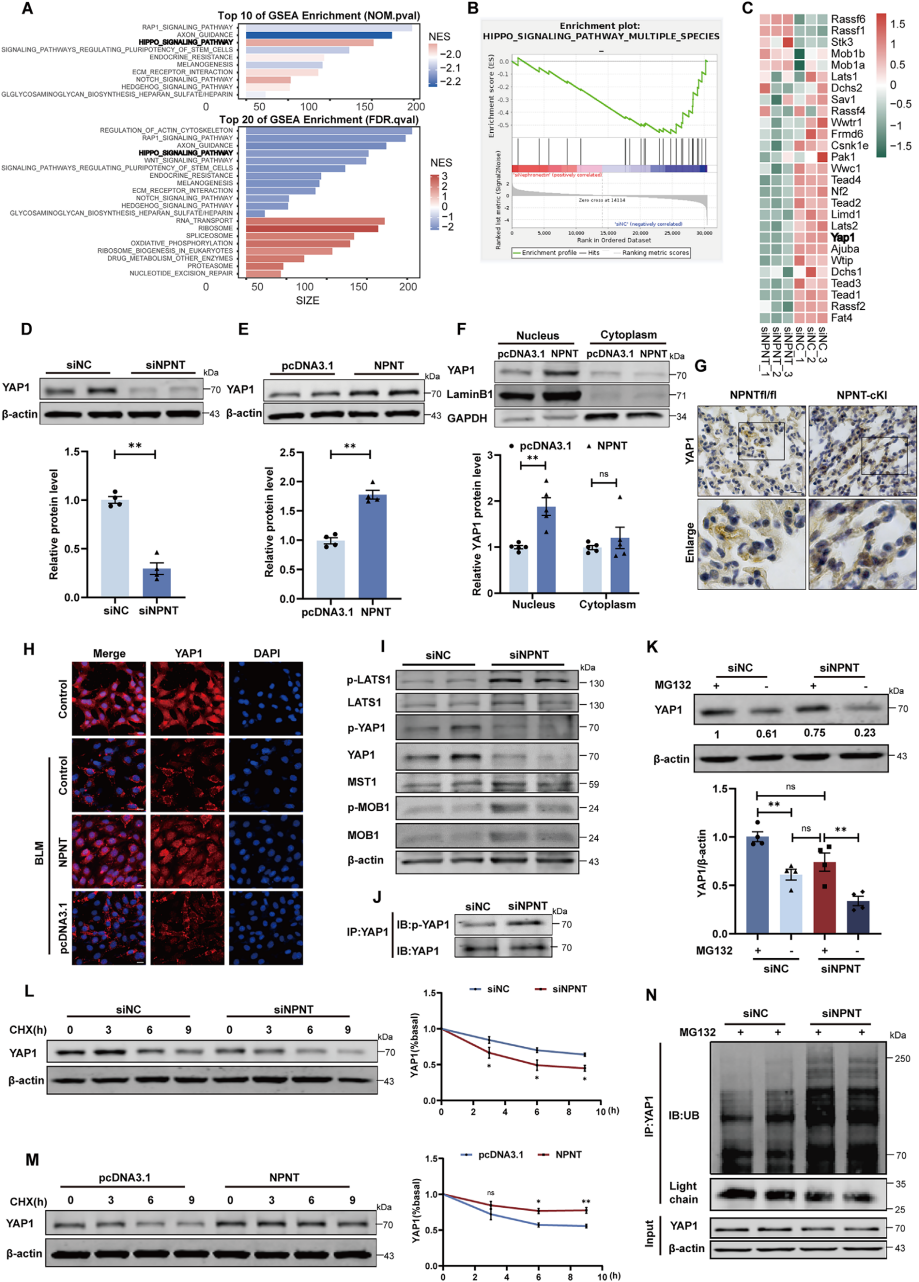
NPNT regulated the Hippo/YAP1 axis in MLE‐12 cells. A) GSEA enrichment analysis of silencing NPNT‐mediated differential genes. B) Enrichment score of the Hippo signaling pathway. C) Transcriptome expression levels of Hippo signaling pathway related genes after siNC and siNPNT transfection (*n* = 3 per group). D,E) Representative diagram of YAP1 protein expression after overexpression and silencing of NPNT, respectively (*n = *4 per group). F) Nuclear/cytoplasmic isolation and Western blot analysis revealed YAP1 translocation. GAPDH and Lamin B were used as cytoplasmic and nuclear markers, respectively (*n = *5 per group). G) Immunohistochemical staining revealed YAP1 expression and cellular localization in the lung tissues of NPNT‐fl/fl and NPNT‐cKI mice. *n = *4 samples per group. Scale bar, 20 µm. H) Representative images taken by confocal fluorescence microscopy showed the intracellular distribution of YAP1 in MLE‐12 cells after different treatments (*n = *4 per group). Scale bar, 20 µm. I) Effect of silencing NPNT on protein expression or phosphorylation of Hippo signaling components. J) YAP1 protein immunoprecipitation was used to measure phosphorylation rate of YAP1. K) MLE‐12 cells transfected with siNC or siNPNT were treated with proteasome inhibitor MG132. The expression of YAP1 was detected by Western blot. Numbers indicated the relative intensity of the band of YAP1 against β‐actin. *n = *4 samples per group. L,M) MLE‐12 cells silencing NPNT or overexpressing NPNT, respectively, were treated with CHX for the indicated time. The expression level of YAP1 was detected by Western blot (*n = *3 per group). N) siNC and siNPNT were transfected in MLE‐12 cells to evaluate the effect of silencing NPNT on YAP1 ubiquitination. Cells were treated with MG132 before harvesting to avoid degradation of ubiquitinated proteins. Data are presented as mean±SEM. ^*^ *p* < 0.05, ^**^ *p* < 0.01.

We further investigated the downstream signaling pathways of NPNT in the aging process of alveolar epithelial cells. RNA sequencing was performed to analyze gene expression in MLE‐12 cells treated with siNPNT versus siNC. GSEA enrichment analysis of differentially expressed genes revealed that silencing NPNT suppressed the Hippo pathway‐related genes (**Figure** **5**A,B). Specifically, several downstream transcription factors of the Hippo signaling pathway, including YAP1, TEAD1, and NF2, were suppressed by siNPNT (Figure 5C). YAP1, an important effector of the Hippo signaling pathway, has been shown in our previous studies to reduce cellular senescence and exert antifibrotic effects when overexpressed in AT2 cells.^[^ ^18^ ^]^ Immunoblotting findings revealed that NPNT promoted the protein level of YAP1, while NPNT knockdown had the opposite effect (Figure 5D,E). Further analysis of nuclear and cytoplasmic proteins showed that NPNT overexpression promoted nuclear translocation of YAP1 (Figure 5F). Compared with NPNT‐fl/fl mice, NPNT‐cKI mice exhibited upregulated YAP1 expression in lung tissues, particularly in terms of nuclear YAP1 expression (Figure 5G). Consistently, NPNT overexpression in MLE‐12 cells could reverse BLM‐induced loss of nuclear YAP1 and restore its nuclear translocation capacity (Figure 5H). Therefore, we hypothesized that NPNT might regulate YAP1 expression and degradation by influencing the phosphorylation of YAP1's upstream kinases. Western blot findings confirmed that silencing NPNT increased the phosphorylation and activation of LATS1 and MOB1, thereby downregulating YAP1 protein level (Figure 5I). Interestingly, phosphorylation of YAP1 at the Ser127 site, an indicator of YAP1 inactivation, was also reduced. We attribute this reduction to the substantial decrease in total YAP1 protein level. To evaluate this hypothesis, we employed immunoprecipitation of YAP1 protein to assess p‐YAP1 level. The results demonstrated an increased phosphorylation rate of YAP1 in cells with NPNT knockdown (Figure 5J). Upon the administration of MG132 to the cells, it was observed that the inhibition of the proteolytic activity of the proteasome complex prevented the reduction in YAP1 protein level that was induced by the silencing of NPNT (Figure 5K). The Cycloheximide (CHX) pulse‐chase assay showed that NPNT knockdown shortened the half‐life of YAP1 and accelerated its degradation, whereas NPNT overexpression exerted the opposite effect (Figure 5L,M). Our findings demonstrated that the ubiquitination level of YAP1 was markedly elevated in cells transfected with siNPNT, suggesting that NPNT plays a crucial role in inhibiting YAP1 degradation through the ubiquitin‐proteasome pathway (Figure 5N). To identify the ubiquitin ligases involved in NPNT regulation of YAP1 degradation, UbiBrowser was used to predict ubiquitin ligases that acted on YAP1 (Figure S5A, Supporting Information). Guo et al.’s experiment confirmed that YAP1 bound to NEDD4L, and NEDD4L mediated the ubiquitination and degradation of YAP1.^[^ ^19^ ^]^ We transfected NPNT siRNA into MLE‐12 cells, and found that silencing NPNT resulted in an increase in YAP1 binding to NEDD4L (Figure S5B, Supporting Information), which may be involved in the degradation process of YAP1 retained in the cytoplasm.

To determine whether YAP1 mediates the inhibitory effect of NPNT on the senescence of alveolar epithelial cells, we added Verteporfin to the cells to suppress YAP1 expression and activity. In the presence of NPNT overexpression, the protein levels of aging‐related markers P21 and P16, as well as cellular of β‐gal activity, were increased by Verteporfin treatment (Figure S6A–C, Supporting Information). NPNT blocked the inhibition of cell proliferation caused by BLM, but this effect was abolished by Verteporfin (Figure S6D, Supporting Information). Consistent with these findings, Verteporfin addition to cells transfected with NPNT overexpression plasmids, increased ROS production and impaired mitochondrial length and morphology (Figure S6E,F, Supporting Information). These data suggest that the antisenescence effect of NPNT is largely due to activation of YAP1.

### 2.6 NPNT Binds to Integrin Receptor ITGA3 to Regulate the Hippo/YAP1 Axis, Thereby Inhibiting the Senescence of Alveolar Epithelial Cells

**Figure 6:**
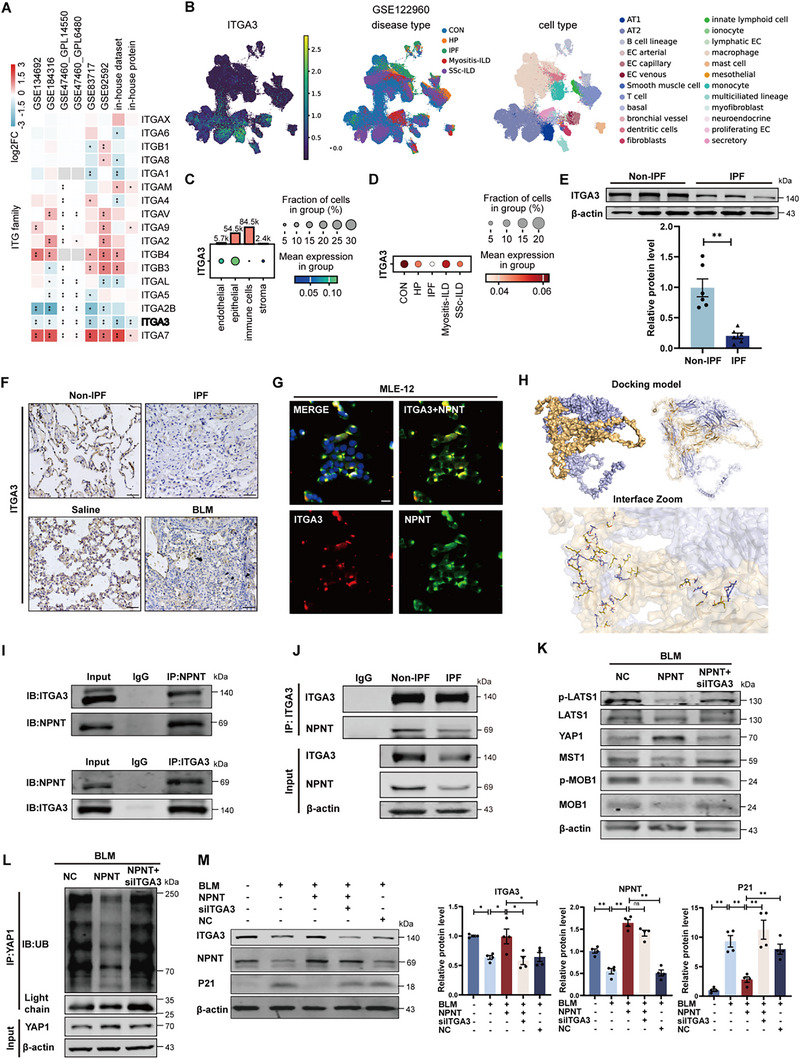
Combination of ITGA3 and NPNT regulates Hippo signal and YAP1 degradation. A) Transcriptome and proteomic analysis of integrin family members in IPF patients versus control group. B–D) Single‐cell dataset was used to analyze the cellular localization of ITGA3 and the expression of ITGA3 in control and IPF patients. E) Western blot and quantitative analysis showed the protein level of ITGA3 in lung tissue of non‐IPF and IPF patients. *n* = 6 samples per group. F) Immunohistochemical staining detection of ITGA3 expression and tissue localization in IPF patients and BLM‐induced pulmonary fibrosis mice (*n = *4 per group). Scale bar, 50 µm. G) Fluorescence co‐localization of NPNT and ITGA3 in MLE‐12 cells. Scale bar, 20 µm. H) Surface diagram of the docking model and its interfacing residues between NPNT and ITGA3 protein (NPNT, orange; ITGA3, blue; hydrogen bond interaction, dotted line). I) Co‐IP analysis of the interaction between NPNT and ITGA3 in MLE‐12 cells. J) Immunoprecipitation of NPNT was performed using anti‐ITGA3 antibodies, and the binding ability of NPNT and ITGA3 between the two groups was detected by Western blotting. K) Representative Western blots showed the protein levels of p‐LATS1, LATS1, YAP1, MST1, p‐MOB1, and MOB1 in MLE‐12 cells. *n = *4 per group. L) ITGA3 was silenced or not under the premise of NPNT overexpression. After BLM induction for 24 h, cells were treated with MG132 and collected. Whole cell lysates were subjected to YAP1 antibody immunoprecipitation and Western blot with anti‐Ubiquitinated antibody to detect YAP1 ubiquitination. M) Representative Western blot bands and statistical data showed the protein level of P21 in siITGA3‐treated cells after NPNT overexpression. *n = *4 per group. Data are presented as mean±SEM. ^*^ *p* < 0.05, ^**^ *p* < 0.01.

To elucidate the receptor responsible for NPNT‐mediated regulation of the Hippo/YAP1 pathway, we focused on the integrin receptor family, given that NPNT is recognized as a ligand for integrin ITGA8. However, studies have shown no significant differences in acute lung injury or fibrosis between ITGA8‐deficient mice and control mice following BLM stimulation,^[^ ^20^ ^]^ suggesting a redundant role for ITGA8 in pulmonary fibrosis. Consequently, we analyzed the expression of integrin receptor family members in the lung tissues of IPF patients and found that both mRNA and protein levels of ITGA3 were significantly downregulated under disease conditions (**Figure** **6**A). Single‐cell dataset analysis revealed that ITGA3 was predominantly enriched in lung epithelial cells, with its expression suppressed in IPF patients (Figure 6B–D). The protein levels of ITGA3 were significantly reduced in lung tissues from IPF patients (Figure 6E,F). Consistent with these findings, mice with BLM‐induced pulmonary fibrosis also exhibited ITGA3 loss (Figure 6F). Notably, ITGA3 was highly expressed in the lungs compared to other tissues (Figure S7A,B, Supporting Information). Therefore, we hypothesized that NPNT might act on ITGA3 to regulate the intracellular Hippo/YAP1 axis, influence the aging of alveolar epithelial cells, and subsequently impact the progression of pulmonary fibrosis. Immunofluorescence results demonstrated spatial colocalization of NPNT and ITGA3 in mouse lung tissues and MLE‐12 cells (Figure 6G; Figure S7C, Supporting Information). Rigid protein docking between NPNT and ITGA3 validated this interaction, with NPNT and ITGA3 forming hydrogen bonds through multiple amino acid residues, indicating a stable protein docking model (Figure 6H). Co‐IP studies confirmed the interaction between NPNT and ITGA3 (Figure 6I). We collected lung tissues from non‐IPF and IPF patients for ITGA3 immunoprecipitation and found a reduced binding affinity of NPNT to ITGA3 in the tissues of IPF patients (Figure 6J). As a driving force, moderate ECM stiffness, known to activate the expansion of epithelial organoids derived from cochlear progenitor cells by modulating the ITGA3/F‐actin cytoskeleton/YAP1 signaling pathway.^[^ ^21^ ^]^ In MLE‐12 cells, NPNT helped maintain cytoskeleton distribution and stability, an effect abolished by ITGA3 silencing (Figure S7D, Supporting Information). In ITGA3‐silenced cells, a notable reduction in nuclear fluorescence intensity of YAP1 was observed, accompanied by an increase in cytoplasmic expression of YAP1 (Figure S7E, Supporting Information). Based on these findings, we simultaneously overexpressed NPNT and silenced ITGA3 in cells. Western blotting showed that NPNT could not effectively suppress the activation of YAP1 upstream kinases and YAP1 degradation after ITGA3 knockdown (Figure 6K,L; Figure S7F, Supporting Information). ITGA3 also mediated the role of NPNT in regulating cell senescence, as evidenced by the fact that the inhibitory effect of NPNT on P21 protein levels was disrupted by siITGA3 (Figure 6M). These data indicate that NPNT binds to ITGA3 on the alveolar epithelial cell membrane, mediating cytoskeleton reorganization and suppressing alveolar epithelial cell senescence by activating the Hippo/YAP1 pathway.

### 2.7 The Protective Effect of NPNT on Pulmonary Fibrosis Mediated by ITGA3

**Figure 7:**
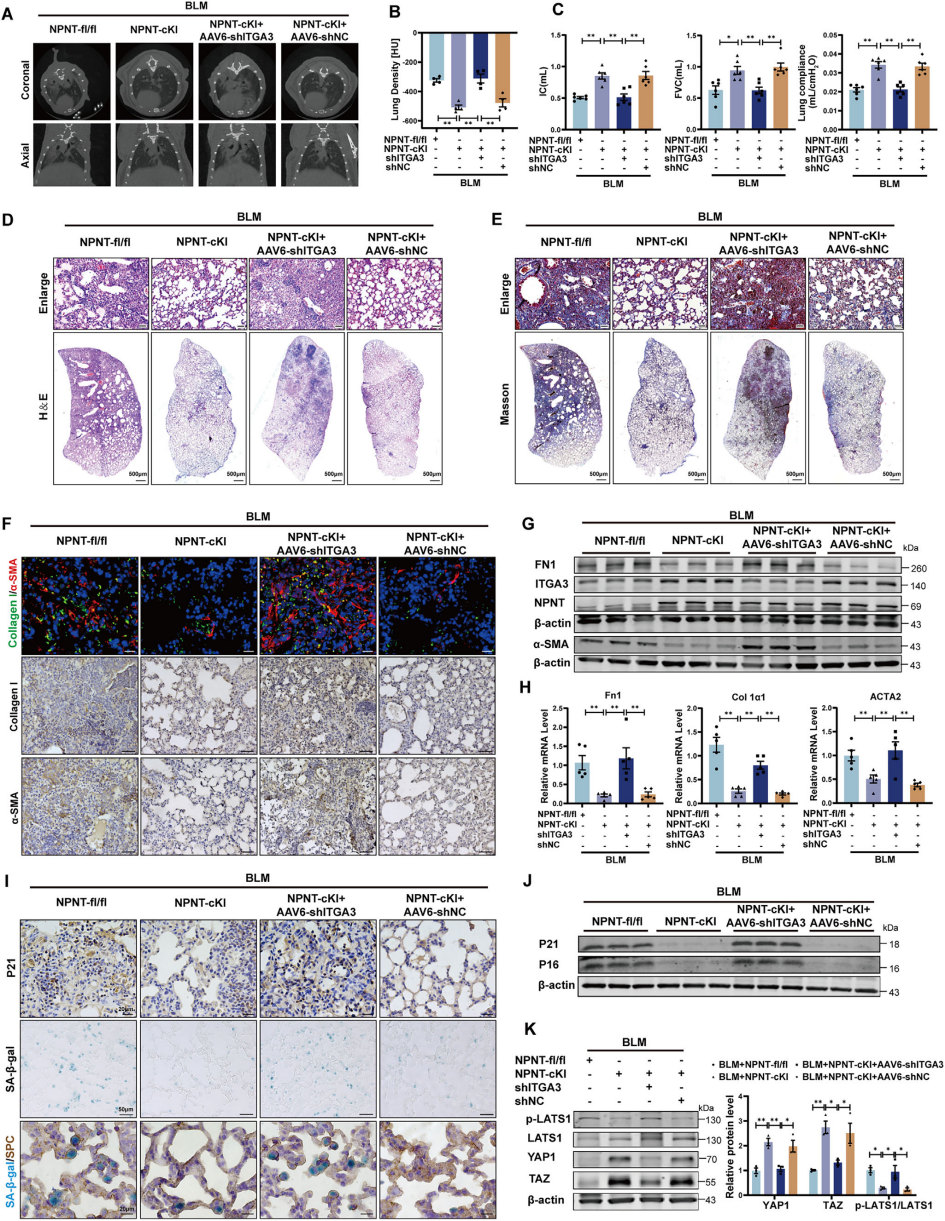
ITGA3 deletion abolished the antifibrotic effect of NPNT overexpression. A,B) Axial and corresponding coronal micro‐CT images were obtained 21 days after BLM administration, and lung density was quantitatively analyzed. *n* = 4. C) The lung function parameters, such as IC, FVC, and lung compliance, were compared among the groups after 21 days of BLM administration. *n = *6 per group. D,E) In the context of BLM‐induced pulmonary fibrosis, H&E and Masson's trichrome staining representative images of lung sections of NPNT‐cKI mice injected with AAV6‐shITGA3 or AAV6‐shNC. *n = *4 per group. Scale bar of upper images, 50 µm. Scale bar of bottom images, 500 µm. F) Immunofluorescence staining (Scale bar, 20 µm) and immunohistochemical staining (Scale bar, 50 µm) images showed the expression of Collagen I and α‐SMA in lung tissues. *n = *4 per group. G) Western blot was used to detect the protein levels of FN1 and α‐SMA in the lung tissue of NPNT‐cKI mice lacking ITGA3. *n = *3 per group. (H) Real‐time quantitative reverse transcription polymerase chain reaction analysis of Fn1, Col 1α1, and ACTA2 mRNA expression in mice treated with BLM. *n = *5 per group. I) SA‐β‐gal and IHC staining images of lung sections from NPNT‐cKI mice treated with AAV6‐shITGA3 or AAV6‐shNC. J) Western blot was used to analyze the protein expression of P21 and P16 in each group of mice after BLM administration. *n = *3 per group. (K) Western blot analysis and statistical data showed that the protein levels of YAP1, TAZ, p‐LATS1, and LATS1 in the lungs of NPNT‐cKI mice were treated with ITGA3 knockdown. *n = *3 per group. Data are presented as mean±SEM. ^*^ *p* < 0.05, ^**^ *p* < 0.01.

This study further investigated whether the protective role of NPNT in pulmonary fibrosis is dependent on ITGA3. In NPNT‐cKI mice, we reduced ITGA3 expression through intratracheal injection of an adeno‐associated virus 6 containing ITGA3 shRNA (AAV6‐shITGA3). Micro‐CT imaging showed that without ITGA3, NPNT failed to reduce the increased lung shadow area and density caused by BLM (**Figure** **7**A,B). Functional testing of lung parameters showed that ITGA3 inhibition exacerbated the decline in IC, FVC, and lung compliance compared with NPNT‐cKI mice (Figure 7C). H&E staining and Masson's trichrome staining revealed that the structural repair of lung tissue and the inhibition of collagen deposition mediated by NPNT overexpression were offset by AAV6‐shITGA3 (Figure 7D,E). Following BLM administration, the expression of ECM‐related proteins and genes, including FN1 and Collagen I, was significantly upregulated in the lungs of NPNT‐cKI mice with ITGA3 knockdown, resulting in increased accumulation of α‐SMA^+^ myofibroblasts (Figure 7F–H).

Moreover, silencing ITGA3 abolished the beneficial effects of NPNT overexpression on lung aging in alveolar epithelial cells. This was specifically manifested by significantly elevated levels of senescence‐related markers P21 and P16, increased SASP secretion, as well as the increased content of β‐galactosidase in lung tissues (Figure 7I,J; Figure S7G, Supporting Information). Western blot analysis demonstrated that the reduction of LATS1 phosphorylation level in NPNT‐cKI mice led to upregulation of YAP1 and TAZ protein expression, which was reversed by ITGA3 shRNA (Figure 7K). These findings indicate that NPNT modulates the Hippo/YAP1 signaling pathway via ITGA3, thereby increasing the protein levels of YAP1.

### 2.8 Escin Improves the Progression of Pulmonary Fibrosis by Maintaining NPNT Protein Expression

**Figure 8:**
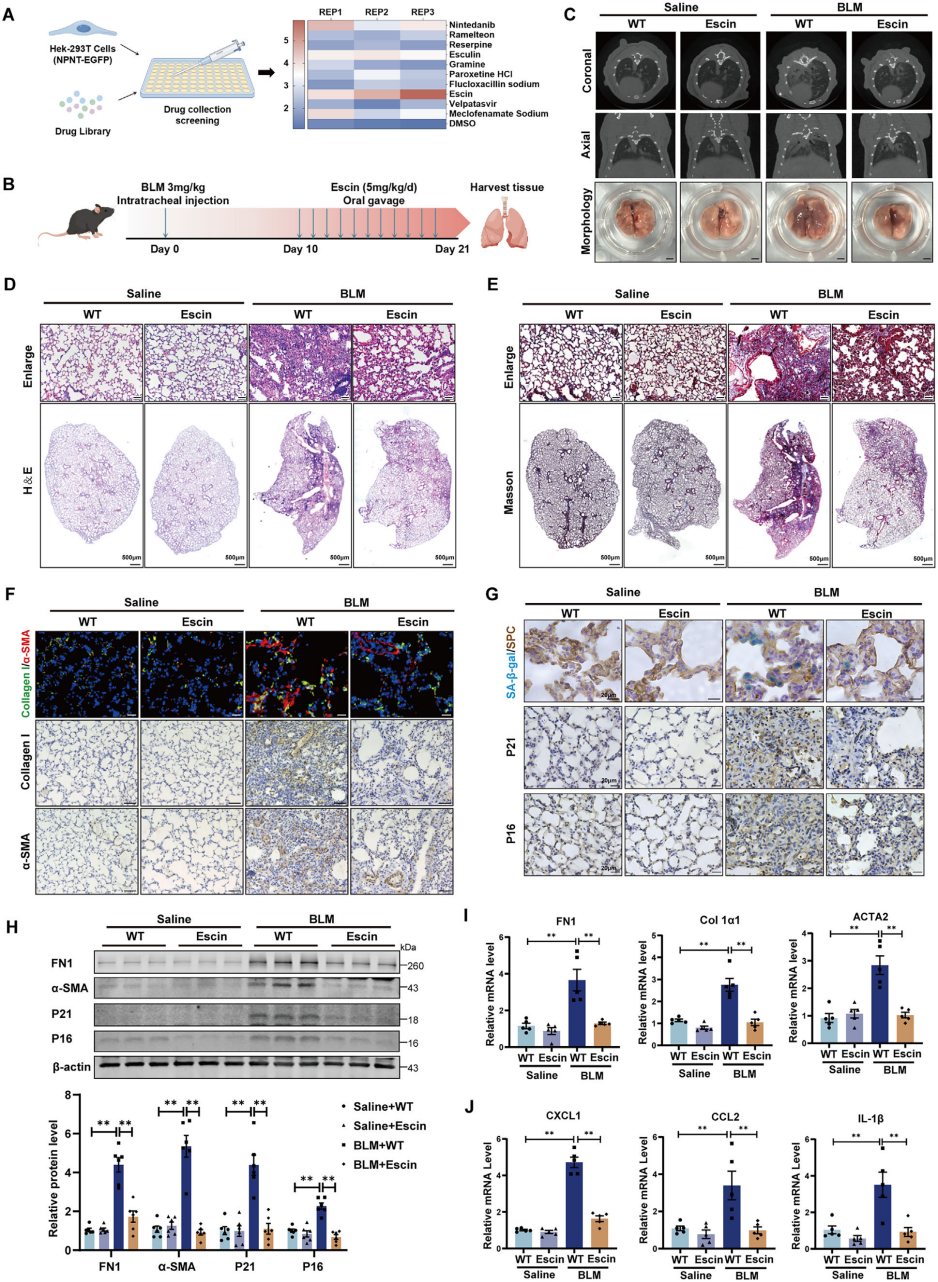
Escin treatment alleviated the progression of pulmonary fibrosis through reducing lung tissue aging. A) Hek‐293T cells expressing NPNT‐EGFP were treated with 3105 compounds, and the fluorescence intensity was detected. The heat map depicted the EGFP intensity of cells treated with ten identified compounds. B) Schematic diagram of Escin treatment in BLM‐induced pulmonary fibrosis mouse model. C) Micro‐CT images displayed the shadow area of the lungs of mice using Escin after BLM administration. *n* = 4 per group. D,E) H&E staining and Masson's trichrome staining of mouse lung sections reflected lung tissue structure and collagen accumulation. Scale bar, 50 µm. F) Representative images of immunofluorescence (Scale bar, 20 µm) and immunohistochemical staining (Scale bar, 50 µm) in lungs of mice treated with or without Escin after BLM induction. *n* = 4 per group. G) Representative images of immunohistochemistry and SA‐β‐gal staining in lungs of mice with or without Escin after intratracheal injection of saline or BLM. *n* = 4 per group. H) Western blot and quantification showing protein levels of FN1, α‐SMA, P21, and P16 in WT and Escin mice 3 weeks after Saline or BLM treatment. *n* = 6 per group. I–J) The mRNA levels of fibrosis‐related markers and inflammatory factors in each group were measured quantitatively. *n* = 5 per group. Data are presented as mean±SEM. ^*^ *p* < 0.05, ^**^ *p* < 0.01.

Our findings suggest that restoring NPNT expression and its signal pathway could be a therapeutic strategy for pulmonary fibrosis. To identify compounds that might promote NPNT expression, we established Hek‐293T cells stably expressing an NPNT‐EGFP fusion protein and screened a library of 3105 compounds from Selleck Chemicals (https://www.selleck.cn/). After a 24h incubation with these compounds, Escin was found to significantly enhance the fluorescence intensity of EGFP‐NPNT (**Figure** **8**A; Figure S8A, Supporting Information). We performed a cell viability assay, and the IC50 of Escin in MLE‐12 cells was 59.47 µm (Figure S8B, Supporting Information). Western blot analysis demonstrated that the addition of Escin (10 µm) to the cells indeed elevated the protein level of NPNT (Figure S8C,D, Supporting Information). Using the fluorescent probe DCFH‐DA, we observed a marked decrease in intracellular ROS levels in the Escin‐treated group compared to the BLM‐stimulated group (Figure S8E, Supporting Information). Therefore, we selected Escin to further investigate its regulatory role in pulmonary fibrosis.

Escin, extracted from horse chestnut (Aesculus hippocastanum) has antiedema and anti‐inflammatory effects. It is used to treat several clinical conditions, including venous insufficiency, pain, inflammation, and edema. On the 10th day following BLM treatment, mice were administered 5 mg kg^−1^ Escin daily via gavage, with lung tissues collected on the 21st day (Figure 8B). Micro‐CT imaging showed that Escin effectively reduced the lung shadow area induced by BLM and improved the lung function in BLM‐treated mice (Figure 8C; Figure S8F,G, Supporting Information). Escin administration helped to stabilize NPNT protein levels and mitigated the loss of NPNT protein caused by BLM (Figure S8H, Supporting Information). In addition, Escin treatment improved the progression of pulmonary fibrosis in mice, characterized by reduced alveolar structural changes and inflammatory cell infiltration (Figure 8D), inhibition of collagen deposition and α‐SMA^+^ myofibroblast production in lung tissues (Figure 8E,F; Figure S8I, Supporting Information), as well as the reduction of lung weight to body weight ratio (Figure S8J, Supporting Information). BLM induced the accumulation of senescent cells in the lungs, whereas Escin treatment significantly reduced the number of P16 and P21 positive cells, specifically suppressing the senescence of SPC^+^ alveolar epithelial cells (Figure 8G). Western blot and qRT‐PCR results further confirmed that Escin reversed the upregulation of fibrosis‐related proteins and genes induced by BLM, such as FN1, Collagen I, and α‐SMA (Figure 8H,I), reduced the protein levels of aging marker genes P16 and P21, as well as suppressed SASP release (Figure 8H,J). Taken together, these data indicate that Escin, by stabilizing and restoring of NPNT protein expression, can effectively reduce fibrosis and lung pathological changes, hence highlighting its potential as a promising therapeutic target for pulmonary fibrosis.

## 3 Discussion

**Figure 9:**
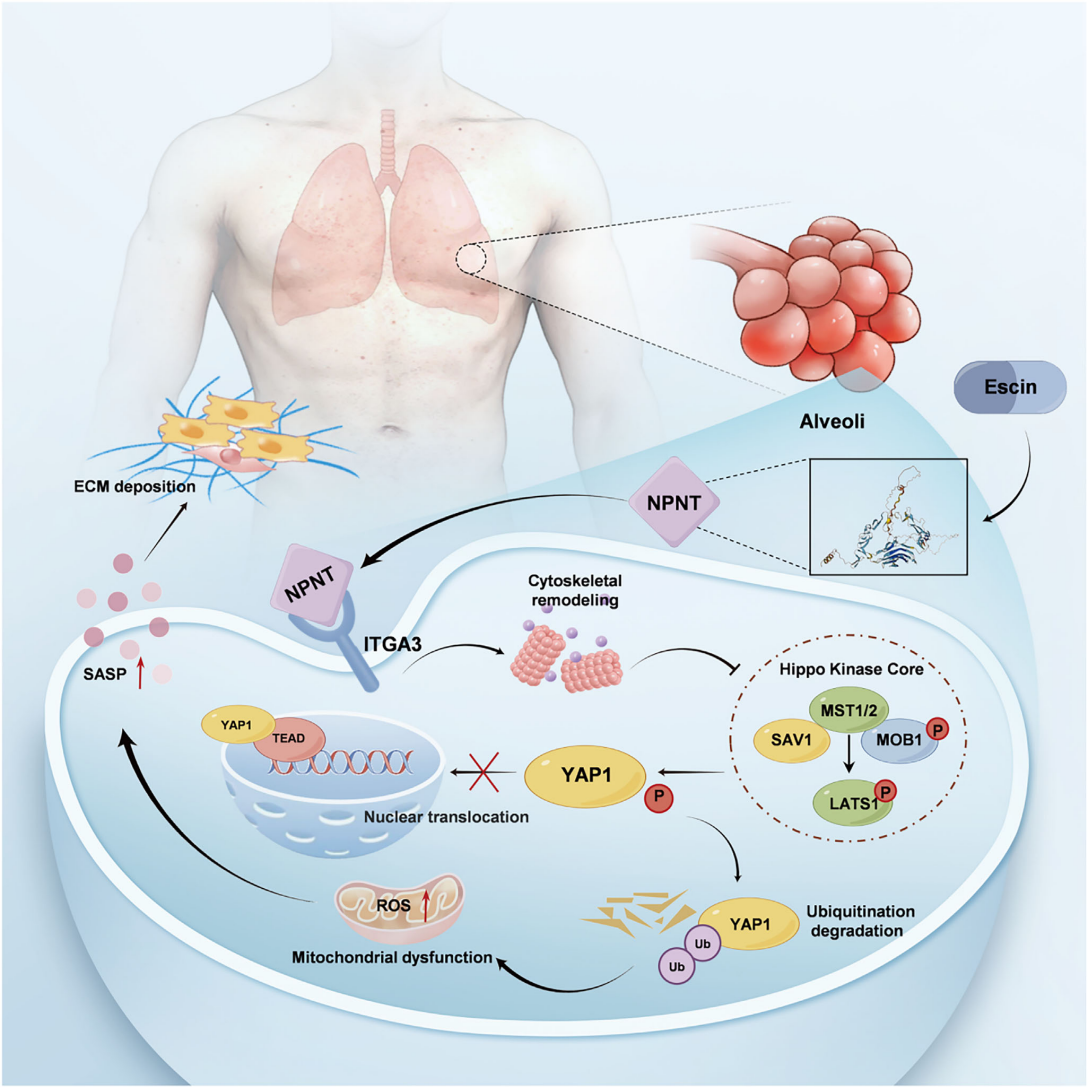
A mechanistic model of NPNT binding integrin receptor ITGA3 mediated Hippo/YAP1 axis in pulmonary fibrosis. In the normal lung, NPNT combines with ITGA3 in alveolar epithelial cells, mediates the polymerization degree of F‐actin, inhibits the excessive activation of Hippo pathway kinase, and is conducive to YAP1 nuclear entry to play the role of a transcription factor. However, in pulmonary fibrosis, NPNT expression is significantly downregulated, which leads to YAP1 retention in the cytoplasm and degradation by ubiquitin proteasome. Inactivation of YAP1 subsequently causes mitochondrial dysfunction and alveolar epithelial cell senescence, releases a large amount of SASP, stimulates ECM deposition and myofibroblast generation, and finally promotes the progression of pulmonary fibrosis.

In this study, we have established that the aberrantly downregulated NPNT in the AT2 cells promotes alveolar epithelial aging and pulmonary fibrosis. This conclusion is supported by an array of evidence, including that NPNT is significantly reduced in both human and mouse fibrotic lungs; this downregulation is inversely correlated with lung function in patients with IPF. In human tissues, NPNT expression is specifically abundant in the lungs and enriched in the lung epithelium. We show here that NPNT suppresses BLM‐induced senescence of lung epithelial cells by inactivating upstream kinase activation of the Hippo signal, maintaining the expression of key effector YAP1. These beneficial effects rely on binding to the integrin receptor α3 (**Figure** **9**). Furthermore, we investigated the role of NPNT signaling in experimental pulmonary fibrosis in mice. NPNT loss promoted alveolar epithelial senescence and aggravated BLM‐induced pulmonary fibrosis, whereas overexpression of NPNT in AT2 cells attenuated the development of pulmonary fibrosis. After screening 3105 compounds, we discovered that Escin could stabilize the protein expression of NPNT, and further alleviate pulmonary fibrosis based on this effect. Therefore, NPNT gene treatment may effectively ameliorate pulmonary fibrosis. Our findings reinforce the current understanding of the ECM‐related gene as a cardinal regulator of pulmonary fibrosis pathologies.

Notably, NPNT is an extracellular matrix protein with five EGF‐like repeats, a mucin region with an RGD sequence, and a COOH‐terminal MAM domain. It can be bound by integrin receptors and several additional RGD‐binding integrins.^[^ ^22^ ^]^ Embryos lacking a functional NPNT gene commonly present with renal agenesis or hypoplasia.^[^ ^23^ ^]^ Other cues have been reported that NPNT promoted cardiac sarcomere maturation and alignment, intercellular communication, as well as synchronous contraction in the heart, with excellent performance in cardiomyocyte adhesion and function.^[^ ^24^ ^]^ Studies have shown that SLIT2 and NPNT are enriched at the embryonic stage and promote cell division of postnatal cardiomyocytes in vitro and vivo.^[^ ^25^ ^]^ Nonetheless, the role and underlying mechanism of NPNT as well as its receptors in the progression of pulmonary fibrosis remain unclear. In this study, our findings showed that the protein and mRNA levels of NPNT were significantly downregulated in the lungs of IPF patients and the BLM‐induced mice pulmonary fibrosis model. Through the construction of NPNT knockdown mice and epithelial‐specific overexpression mice, combined with cell experiments, we obtained strong evidence that NPNT regulated the occurrence and development of pulmonary fibrosis by affecting epithelial cell senescence. Targeting other ECM proteins (e.g., laminins, fibronectin) risks disrupting basement membrane homeostasis or interfering with early repair processes,^[^ ^26^, ^27^ ^]^ posing significant clinical safety concerns. In contrast, targeting the NPNT‐ITGA3‐YAP axis may enable precise modulation of epithelial cell function while avoiding off‐target effects associated with broad ECM regulation. NPNT has the capacity to inhibit the senescence of AT2 cells, partially restore cellular proliferation, and facilitate the repair of lung tissue. Concurrently, NPNT relieves the ECM remodeling, disrupts the core processes of fibrosis, and reduces lung tissue stiffness. This mechanism of action is distinct from approaches that directly eliminate senescent cells (e.g., Senolytics) or regulate metabolism (e.g., NAD+ precursors).^[^ ^28^, ^29^ ^]^ Future research should investigate the potential synergistic effects of NPNT in conjunction with other anti‐aging therapies, as this combination may offer a promising treatment strategy for pulmonary fibrosis.

Integrins are transmembrane αβ glycoproteins connecting the extracellular matrix and cytoskeleton as well as the most important receptors for ECM proteins.^[^ ^30^, ^31^ ^]^ Integrin receptors have been widely used in clinical practice, especially in tumor diagnosis and treatment, inflammatory diseases, and cardiovascular diseases. NPNT was initially identified as an extracellular matrix ligand for integrin α8β1.^[^ ^22^ ^]^ Following TGF‐β treatment, pulmonary PDGFRβ^+^ stromal cells with ITGA8 deficiency exhibited increased Col1a1 mRNA expression; however, no functional differences were observed in vivo,^[^ ^20^ ^]^ suggesting a redundant role of ITGA8 in pulmonary fibrosis. ITGA8 deletion did not significantly affect lung fibrosis progression but exacerbated renal fibrosis, highlighting its organ‐specific functional divergence.^[^ ^32^ ^]^ In contrast, ITGA3 is highly expressed in lung tissue and alveolar epithelial cells, which has attracted our attention. Human ITGA3 mutations cause congenital nephrotic syndrome, epidermolysis bullosa, and interstitial lung disease, also known as NEP syndrome.^[^ ^33^ ^]^ Alanine to serine substitution and ITGA3 glycosylation mutation of the integrin α3 subunit prevent the biosynthesis of functional α3β1 and cause fatal interstitial lung disease.^[^ ^34^ ^]^ These suggest that the ITGA3 gene may have important significance in the treatment and diagnosis of respiratory diseases. Of note, moderate ECM stiffness acts as a driving force to activate the expansion of epithelial organoids by regulating ITGA3/F‐actin cytoskeleton/YAP1 signaling.^[^ ^21^ ^]^ The pathway activated by ITGA3 modulates YAP1 phosphorylation and nuclear localization via FAK/CDC42/PP1A cascade signaling, coordinating stem cell expansion and differentiation in organ renewal.^[^ ^35^ ^]^ However, as a transcriptional coactivator, the Hippo pathway effector YAP1 could form a complex with ZEB1 to activate ITGA3 transcription through YAP1/TEAD binding sites.^[^ ^36^ ^]^ Integrin receptors and the Hippo pathway form a tight network through mechanical signaling, cytoskeletal dynamics, and molecular interactions to co‐regulate cell fate. Integrins activate the RhoA/ROCK signaling pathway, promoting actin filament polymerization and stress fiber formation. Increased cytoskeletal tension inhibits LATS kinase, thereby activating YAP/TAZ. Additionally, within integrin‐clustered focal adhesions, FAK and ILK regulate the subcellular localization of YAP/TAZ through phosphorylation events.^[^ ^37^, ^38^ ^]^ In this study, ITGA3 and NPNT were colocalized and effectively bound in lung epithelial cells, which was the basis for promoting cytoskeletal remodeling and YAP1 nuclear translocation. Using an adeno‐associated virus 6 targeting the lung epithelium to ITGA3 knockdown, we found that ITGA3 deficiency eliminated the improvement of NPNT on cell aging and pulmonary fibrosis. These indicate that NPNT regulates the Hippo/YAP1 signaling axis by affecting cytoskeleton remodeling through binding to ITGA3 receptors, hence highlighting the important role of ITGA3 in mediating NPNT antipulmonary fibrosis.

The Hippo signaling pathway regulates cell proliferation and differentiation, organ size control, as well as the sensing and transmission of mechanical force. YAP1 is a major effector of Hippo signaling, and its role in cellular senescence is broadly recognized. Maintaining the role of YAP1 can rejuvenate senescent cells and oppose the appearance of senescence characteristics associated with physiological senescence or accelerated senescence induced by mechanically defective extracellular matrix.^[^ ^39^ ^]^ Our previous work has revealed that mice overexpressing YAP1 in alveolar epithelial cells can enhance mitochondrial dysfunction, suppress cellular senescence, and alleviate the progression of pulmonary fibrosis.^[^ ^18^ ^]^ Herein, our findings revealed that the Hippo/YAP1 pathway plays a key role in NPNT inhibition of alveolar epithelial cell senescence. NPNT deletion promoted phosphorylation and activation of LATS1 and MOB1, the upstream kinases of Hippo, as well as prevented YAP1 from translocating to the nucleus. YAP1 residing in the cytoplasm was ubiquitinated and degraded by E3 ubiquitin ligases (e.g., NEDD4L), thereby losing its function as a transcription factor. Verteporfin, a YAP1 inhibitor, abolished the protective effect of NPNT on alveolar epithelial cell senescence and maintenance of mitochondrial homeostasis, and increased cellular ROS production. The results highlight that NPNT prevents cellular senescence by modulating the Hippo pathway, influencing YAP1 degradation, and nuclear translocation.

Escin is a natural product of triterpenoid saponins that can be isolated from Aesculus seed and can be used as an active molecule for vascular protection, anti‐inflammatory, anti‐edema, and anti‐injury. Escin can prevent and treat venous edema and tissue edema by reducing vascular permeability and resisting exudation, thereby alleviating venous congestion. Escin activates ALDH to inhibit smoking‐induced cell death, and alleviates non‐alcoholic fatty liver disease through activating antioxidants and autophagy via Keap1‐Nrf2.^[^ ^40^, ^41^ ^]^ β‐escin has a repair effect on atrophic regenerated muscle, it reduces inflammatory infiltration and fibrosis, as well as increases the number of muscle fibers.^[^ ^42^ ^]^ However, there is no evidence for the role of Escin in pulmonary fibrosis. In our study, Escin increased the expression of NPNT and prevented NPNT loss during pulmonary fibrosis. Herein, we investigated the protective effect of Escin on pulmonary fibrosis, and found that it effectively reduced the number of senescent epithelial cells, inhibiting extracellular matrix deposition and SASP gene expression, which is conducive to the recovery of lung function, and can provide a valuable reference for possible clinical trials in the future, and provide a new drug and target for clinical prevention and treatment of pulmonary fibrosis.

This study revealed for the first time that NPNT binds to the integrin receptor ITGA3, regulates the Hippo/YAP1 signaling pathway by modulating the epithelial cytoskeleton remodeling, and participates in the occurrence of cellular senescence and pulmonary fibrosis, which explains the association between NPNT and lung function. However, there are some limitations that need to be addressed. Heterozygous NPNT+/− mice may mask phenotypes due to residual gene expression, such as subthreshold effects, while conditional knockout enables gene deletion specifically in lung AT2 cells or at particular developmental stages, circumventing lethal phenotypes and avoiding compensatory interference from other organs. Second, the exact molecular link between NPNT binding to ITGA3 and LATS1/MOB1 inactivation still needs to be explored in detail. Although the MLE‐12 cell line is widely used, it has certain limitations. As a monolayer‐cultured 2D cell model, it cannot fully recapitulate the complex microenvironment of alveolar tissue in vivo. Considering the secretory function of extracellular matrix proteins, it remains to be determined whether NPNT may be involved in other cell types via the paracrine action and participate in pulmonary fibrosis.

## 4 Experimental Section

### Human Subjects

Human IPF lung tissue was derived from organ transplant patients in Shanghai Pulmonary Hospital. Informed consent of the patients was obtained, and the study was approved by the Ethics Committee of the Shanghai Pulmonary Hospital (No. K24‐015) and in accordance with the Declaration of Helsinki.

### Animal Experimental Protocols

All animal experiments were performed in accordance with the guidelines and with the approval of the ethics committee of the School of Pharmacy, Harbin Medical University (IRB2023724). NPNT‐fl/fl and Sftpc‐CreERT2 mice were purchased from Cyagen Biosciences (Guangzhou, China), and wild‐type C57BL/6 mice (22–25 g) were purchased from Liaoning Changsheng Biotechnology Co., Ltd. (Benxi, China). NPNT‐fl/fl was crossed with Sftpc‐CreERT2 mice to generate NPNT‐fl/fl; Sftpc‐CreERT2 transgenic mice, hereinafter referred to as NPNT‐cKI mice. The transgenic mice were treated with 75 mg kg^−1^ tamoxifen for 7 days and then rested for more than 7 days to avoid adverse reactions. NPNT knockout mice were derived from Cyagen Biosciences, and since the reproductive rate of NPNT homozygous mutant (NPNT−/−) mice is much lower than Mendelian laws of inheritance, heterozygous NPNT+/− mice were used for all experiments. Male 8–12‐week‐old mice were used at the beginning of the experiment and genotyped by PCR. Animals were randomly assigned and anesthetized. To establish a pulmonary fibrosis model, mice were given 3 mg kg^−1^ BLM intratracheally, and their lungs were harvested 21 days later. For mice in the Escin treatment group, after a single intratracheal injection of BLM, oral gavage administration of Escin (5 mg kg^−1^ day^−1^, Selleck, 6805‐41‐0, 100.00% purity) was initiated on day 10 post‐BLM instillation and continued consecutively for 10 days. The mice were euthanized by cervical dislocation to ensure the death of the animals.

### Cell Culture

MLE‐12 cells were purchased from Shanghai Huzhen Real Industry Go. Ltd., cultured in DMEM/F‐12 (Sigma‐Aldrich, Michigan, USA) supplemented with 10% bovine serum and 1% penicillin‐streptomycin. All cells were in a humidified atmosphere at 37 °C, 5% CO_2_. Human NPNT overexpression plasmid and control vector pcDNA3.1, mouse NPNT siRNA (siNPNT) and nontarget control siRNA (siNC) constructs were purchased from Gene Create (Wuhan, China). During transfection, DMEM/F‐12 was used to replace the whole medium. Next, plasmids or siRNAs were mixed with Lipofectamine 2000 (Life Technologies, California, USA) and opti‐MEM (Invitrogen, USA), added to the well plates, and incubated for 6 h for subsequent experiments.

### Micro‐CT Analysis

The mice were anesthetized and transferred to a Quantum GX II scanner (PerkinElmer, Houston, USA) to scan the lung area. The scanning conditions were as follows: the X‐ray tube was set at 70 kV, 80 µA, the pixel size was 50 µm, the field of view (FOV) was scanned at 25 mm, and the scanning time was 4 min to allow high‐resolution imaging of mice. Images were automatically generated by AutoViewer and post‐processed by relevant system software to evaluate the different degrees of pulmonary fibrosis.

### Lung Function Measurements

According to the manufacturer's protocol, DSI Buxco PFT Controller Pulmonary Function Testing was used for invasive lung function testing. Briefly, anesthetized mice were intubated and connected to a computer‐controlled ventilator system. The inspiratory gas flow rate was set to fill the lungs of mice with gas within 2–3 s, and the slow expiratory flow rate was set to exhaust the lung capacity of mice as much as possible within a given time. The lung function parameters, such as inspiratory capacity, forced vital capacity, and lung compliance, were recorded with the acquisition software FinePointe.

### Histopathology and Immunohistochemistry

Mouse lung specimens were fixed with 4% paraformaldehyde (Solarbio, Beijing, China), dehydrated, and embedded in paraffin, and cut into 5 µm thick sections. Tissue sections were dewaxed with xylene and rehydrated with graded ethanol. After incubation with 3% hydrogen peroxide, citrate buffer was microwave‐heated to repair the antigen and cooled to room temperature. Sections were blocked with normal goat serum albumin for 1 h. Lung sections were incubated with primary antibodies overnight at 4 °C. The next day, the lung tissue was incubated with secondary antibodies, stained with DAB (ZSGB‐BIO, Beijing, China), and the nuclei were stained with hematoxylin. The following primary antibodies were used: anti‐α‐SMA (1:300, AF1032, Affinity Biosciences, Jiangsu, China), anti‐Collagen I (1:300, AF7001, Affinity Biosciences, China), anti‐P21 (1:200, WL0362, Wanlei, Shenyang, China), anti‐P16 (1:200, WL01418, Wanlei, Shenyang, China), anti‐NPNT (1:100, sc‐393033, Santa Cruz, Dallas, USA), anti‐ITGA3 (1:300, 21992‐1‐AP, Proteintech, Wuhan, China). For H&E staining and Masson's trichrome staining, lung sections were stained using Masson's staining or H&E staining kit purchased from Solarbio Life Sciences (Beijing, China).

### Immunofluorescence Staining

Lung tissues were embedded with OCT (Solarbio, China) and prepared into 6 µm thick sections. After frozen sections were fixed with acetone, 3% H_2_O_2_ blocked endogenous peroxidase. Normal goat serum was blocked for 1 h at room temperature, then incubated with primary antibody at 4 °C overnight. The next day, Alexa Fluor 488 conjugated Goat antirabbit IgG (1:500, Proteintech, China) and Alex fluor 594 conjugated Goat antimouse IgG (1:500, Proteintech, China) were treated for 1 h at room temperature, and DAPI was used for nuclear staining. Images were acquired using a Zeiss confocal microscope.

### Hydroxyproline Content

The lung tissue samples were accurately weighed, and the hydroxyproline content was determined using the alkaline hydrolysis kit of Nanjing Jiancheng Bioengineering Institute (Nanjing, China). After adjusting the pH, the supernatant was taken, and the absorbance value at 550 nm was measured.

### Senescence‐Associated β‐Galactosidase (SA‐β‐gal) Activity Measurement

The SA‐β‐gal activity was determined with a commercial kit (Beyotime Biotechnology, Shanghai, China) according to the manufacturer's instructions. Briefly, frozen sections of lung tissue or MLE‐12 cells treated as instructed are fixed and incubated with staining buffer at 37 °C for 24 h. At the end of incubation, observe with a 200X magnifying glass.

### Measurement of Intracellular ROS

Intracellular ROS production was determined according to the manufacturer's protocol. Briefly, cells were removed from the culture medium and incubated with DCFH‐DA (Beyotime Biotechnology, China) at a final concentration of 10 µm for 20 min at 37 °C. After that, the cells were washed three times with buffer according to the instructions, and the nuclei were labeled by Hoechst. The fluorescence intensity was photographed and analyzed by a fluorescence microscope.

### Immunoblotting Analysis

Lung tissues or cells were lysed in precooled RIPA lysis buffer containing protease or phosphatase inhibitors (Roche). Protein concentration was determined by the BCA method. Equal amounts of protein (40–60 µg) were loaded into 10%‐12.5% SDS‐PAGE gel electrophoresis for separation, and then the protein was transferred to nitrocellulose membranes (Pall Life Sciences, USA), and blocked with 5% skim milk. Membranes were then incubated with primary antibodies against FN1(1:1000, P02751, Bioworld, Nanjing, China), α‐SMA (1:1000, AF1032, Affinity, China), NPNT (1:200, sc‐393033, Santa cruz, USA), β‐actin (1:1000, 66009‐1‐Ig, Proteintech, China), ITGA3(1:1000, 21992‐1‐AP, Proteintech, China), P21(1:1000, ab188224, Abcam, Cambridge, UK), P16(1:1000, ab189034, Abcam, UK), YAP1(1:1000, 66900‐1‐Ig, Proteintech, China). Hippo pathway‐related proteins were detected using the Hippo Signaling Antibody Sampler Kit (1:1000, 8579T, Cell Signaling Technology, Boston, USA). The next day, the secondary antibody (1:8000, LI‐COR, Nebraska, USA) was incubated for 1 h at room temperature, and the protein band density was quantified with Odyssey image studio version 5.2.

### Co‐Immunoprecipitation

MLE‐12 cells were lysed by RIPA buffer and incubated with antibody conjugated to protein A/G magnetic beads (MCE, Shanghai, China) overnight at 4 °C. Coprecipitating proteins were identified by SDS‐PAGE followed by immunoblotting.

### Quantitative Real‐Time PCR Analysis (qRT‐PCR)

Total RNA was extracted from lung tissue or cells using TRIzol reagent (Invitrogen, California, USA), followed by reverse transcription using ReverTra Ace qPCR RT kit (Code No.FSQ‐101, Japan) was performed. The above cDNA was subjected to qRT‐PCR using SYBR GREEN (Roche, Basel, Switzerland) according to the manufacturer's protocol. All data were normalized to a control group using ACTB as an internal control. Data were analyzed using the comparative Ct (2^−ΔΔCt^) method.

### Mito‐Tracker Analysis

After treatment, cells were incubated with Mito‐Tracker Red CMXRos probes (Beyotime Biotechnology, China) at 37 °C for 30 min in a cell incubator, and then incubated with Hoechst for 10 min, washed thoroughly with buffer, and images were obtained using a confocal microscope.

### Statistical Analysis

All data are presented as mean ± SEM. GraphPad Prism version 8.0 was used for statistical analysis. Statistical analysis students’ *t*‐test and one‐way analysis of variance (ANOVA) were used to determine statistical significance, and Bonferroni corrections were used for multiple comparisons. *p *< 0.05 was statistically significant.

## Conflict of Interest

The authors declare no conflict of interest.

## Author Contributions

J.G., Y.W., Q.L., and Z.L. contributed equally to this work. H.L. and Y.G. conceived the study and designed the experiments. J.G., Y.W., and Q.L. contributed to the methodology and performed data analysis; Z.L., X.T., Y.L., Y.G., and H.G. conducted cellular and molecular biological experiments; Y.W. and Z.N. contributed to data curation and formal analysis; X.W., J.F., M.L., and D.S. performed the animal studies and analyzed the data; Y.Z. and T.P. conducted single‐cell data analysis; Y.B. performed quantitative data sorting of CT scans. T.B. and T.L. oversaw project administration. All authors contributed to the original draft, editing, and revision. All authors reviewed and approved the manuscript.

## Supporting information



Supporting Information

## Data Availability

The data that support the findings of this study are available from the corresponding author upon reasonable request.
