# Supplementary material for: Nephronectin (NPNT) is a Crucial Determinant of Idiopathic Pulmonary Fibrosis: Modulating Cellular Senescence via the ITGA3/YAP1 Signaling Axis
Source: Adv Sci (Weinh). 2025 May 30;12(32):e01956. doi: 10.1002/advs.202501956 (PMC12407361; doi:10.1002/advs.202501956)
Supplement: Supplementary file 1 — Supporting Information [file ADVS-12-e01956-s001.docx]

**Table S1.** The siRNA sequences.

| **siRNA** | **Sense Seq** | **Antisense Seq** |
| --- | --- | --- |
| siRNA-NPNT-1 | CCAUAUGCCAGAAAGAAAU | AUUUCUUUCUGGCAUAUGG |
| siRNA-NPNT-2 | GCAAAUGUCACCCUGGAUU | AAUCCAGGGUGACAUUUGC |
| siRNA-NPNT-3 | GCCUGUCCUUUAGGCACAA | UUGUGCCUAAAGGACAGGC |
| siRNA-ITGA3 | GGACAAUGUUCGCGAUAAACUTT | AGUUUAUCGCGAACAUUGUCCTT |
| Negative control | UUCUCCGAACGUGUCACGU | ACGUGACACGUUCGGAGAA |

**Table S2.** The primers sequences.

| **Gene** | **Forward primer** | **Reverse primer** |
| --- | --- | --- |
| Mus ACTB | 5′-GTGACGTTGACATCCGTAAAGA-3′ | 5′-GCCGGACTCATCGTACTCC-3′ |
| Mus NPNT | 5′-TGAAGCCTCGGCCCTGTAA-3′ | 5′-AGCATGTATCCGTTGAGACAGTA-3′ |
| Mus Col1a1 | 5′-TGGTCCTGATGGCAAAACCG-3′ | 5′-GAATCCCATCACACCAGCCT-3′ |
| Mus Fn1 | 5′-ACCAGTGTTACTGCTACGGC-3′ | 5′-TGGTGATGTGTGAAGGCTCC-3′ |
| Mus ACTA2 | 5′-GTACCACCATGTACCCAGGC-3′ | 5′-GCTGGAAGGTAGACAGCGAA-3′ |
| Mus CCL2 | 5′-CCTGCTGCTACTCATTCACCA-3′ | 5′-ATTCCTTCTTGGGGTCAGCA-3′ |
| Mus IL-1β | 5′-ATGCCACCTTTTGACAGTGATG-3′ | 5′-TGTGCTGCTGCGAGATTTGA-3′ |
| Mus CXCL1 | 5′-TGCACCCAAACCGAAGTCAT-3′ | 5′-ACTTGGGGACACCTTTTAGCA-3′ |
| Hsa ACTB | 5′-CATGTACGTTGCTATCCAGGC-3′ | 5′-CTCCTTAATGTCACGCACGAT-3′ |
| Hsa NPNT | 5′-GGGAGCTACATCTGCAAGTGT-3′ | 5′-CAAGTGAGCATTCGTCTATGTCA-3′ |

**Figure S1**

**
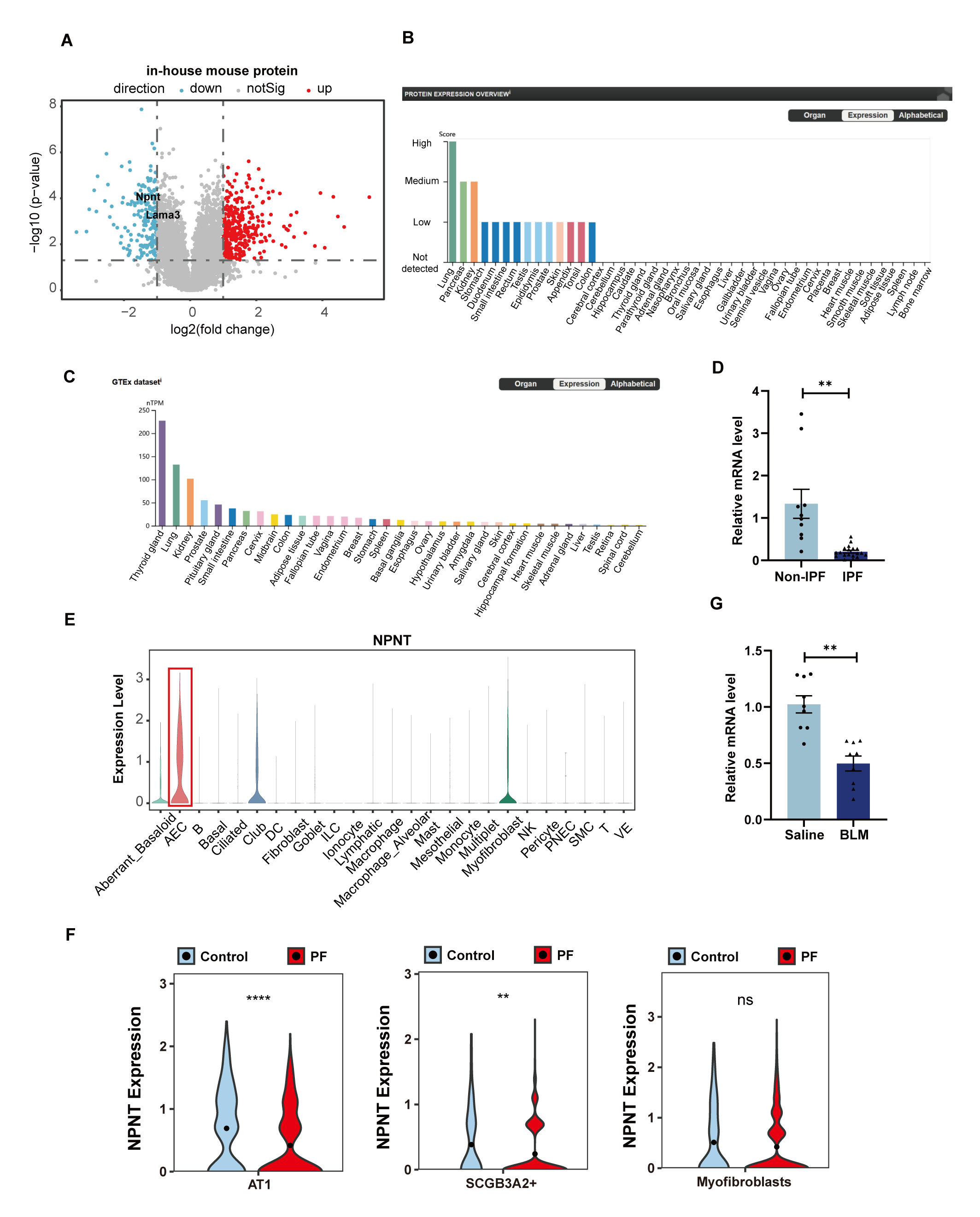
**

**Supplemental Figure S1.** **Tissue enrichment and differential expression of NPNT in pulmonary fibrosis samples.**

(A) Proteomic data of BLM-induced pulmonary fibrosis in mice. n=3 per group. (B) Protein expression overview of NPNT in various tissues. Source of Data: The Human Protein Atlas (https://www.proteinatlas.org/). (C) RNA expression overview of NPNT in tissues. Source of Data: The Human Protein Atlas. (D) Quantitative analysis of NPNT mRNA levels in lung tissues of control group and IPF patients. n=10~19. (E) Expression of NPNT in single-cell transcriptome data from human lung tissue samples in GSE136831. (F) Comparison of NPNT expression in different cells between the control group and PF group in the GSE135893 dataset, evaluated using a two-sided Wilcoxon rank-sum test (***P* < 0.01, *****P* < 0.0001). (G) Quantification of mRNA levels of NPNT in WT mice 3 weeks after Saline or BLM administration. n=9. Data are presented as mean±SEM. ***P* < 0.01.

**Figure S2**


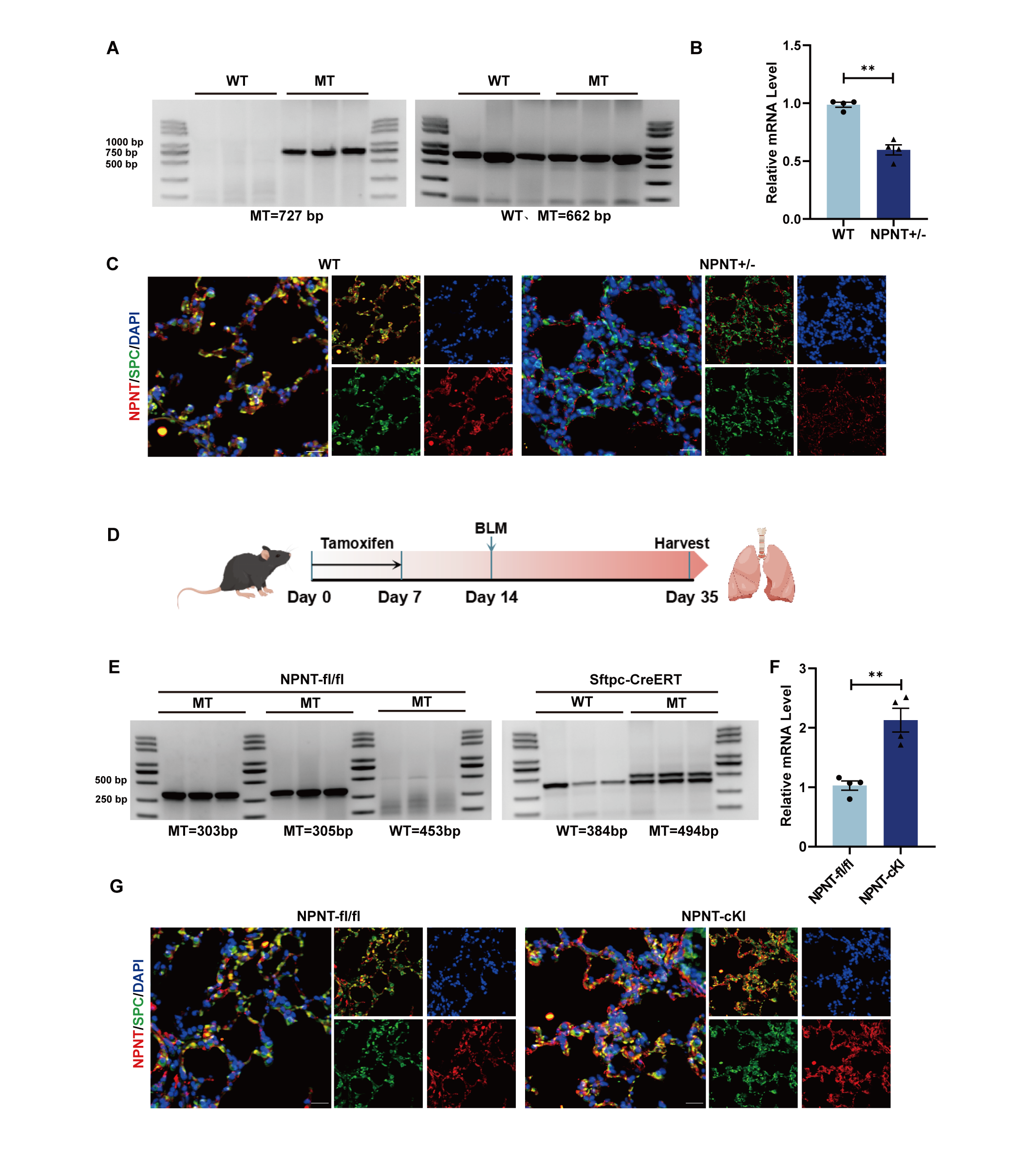


**Supplemental Figure S2**. **Identification of NPNT+/- and NPNT-cKI transgenic mice.** (A) Representative agarose gel diagram showed that the bands of NPNT+/- mutant were 727 bp and 662 bp, while the bands of WT type were only 662 bp. n=3 per group. (B) qRT-PCR quantification of knockout efficiency in NPNT+/- mice. n=4 per group. (C) The fluorescence intensity of NPNT and SPC in WT and NPNT+/- mouse lung sections were detected by immunofluorescence staining. Scale bar, 20μm. (D) Schematic diagram of strategies for constructing pulmonary fibrosis model in transgenic mice with specific overexpression of NPNT in AT2 cells. (E) Representative agarose gel diagram of the mutant type bands of NPNT-fl/fl at 303 bp and 305 bp, Sftpc-CreERT at 494 bp. n=3 per group. (F) Quantification of NPNT mRNA levels in NPNT-fl/fl and NPNT-cKI mice. n=4 per group. (G) Representative images of immunofluorescence staining of NPNT and SPC. n=4 per group. Scale bar, 20μm. Data are presented as mean±SEM. ***P* < 0.01.

**Figure S3**


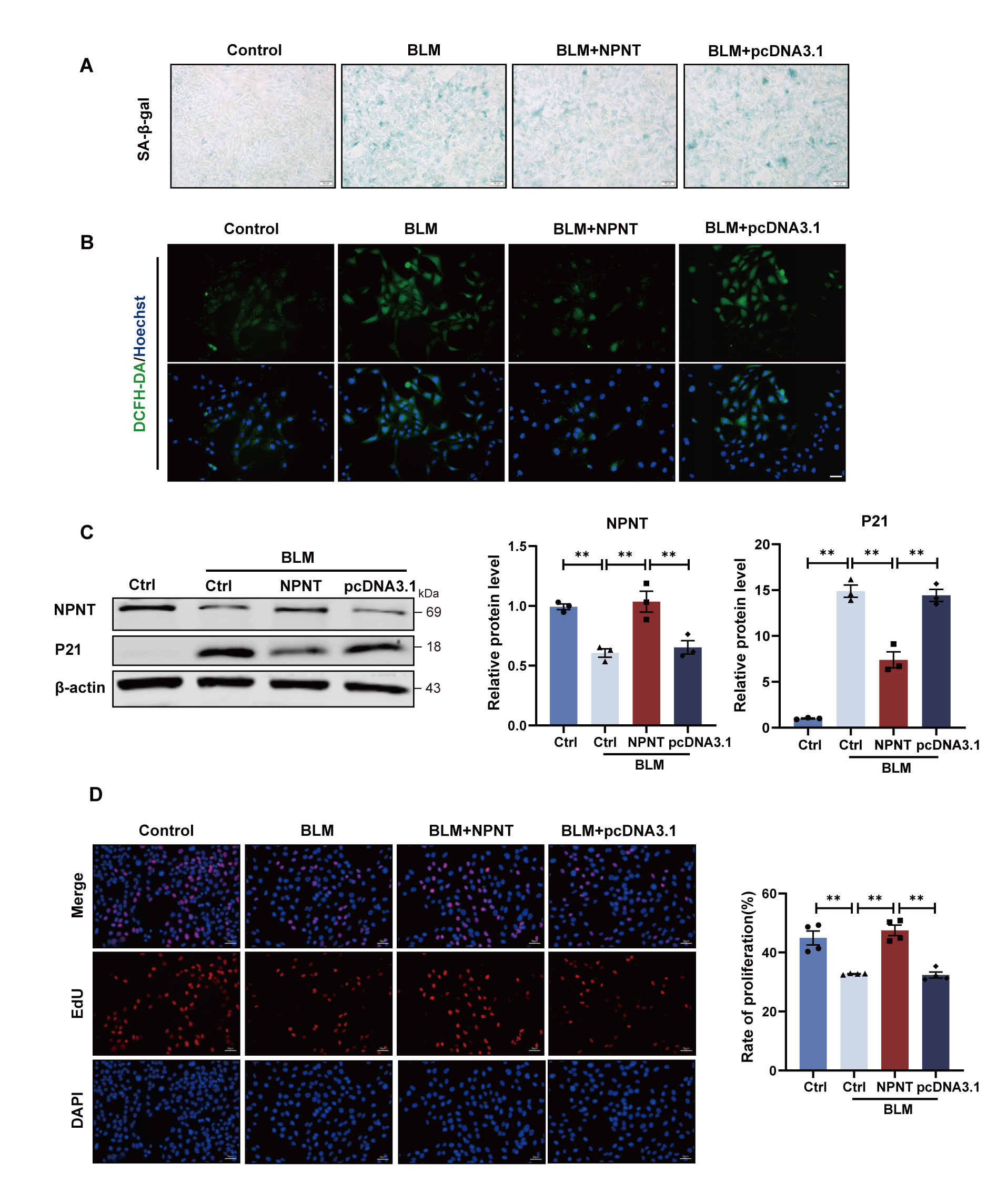


**Supplemental Figure S3. The overexpression of NPNT impeded the senescence process in HPAEpiC-II cells.** (A) SA-β-gal staining to evaluate senescence in HPAEpiC-II cells transfected with the NPNT overexpression plasmid or pcDNA3.1 after BLM treatment. n=4. Scale bars, 50μm. (B) The ROS levels in HPAEpiC-II cells overexpressing NPNT after BLM treatment were labeled with DCFH-DA probe. n=4 samples per group. Scale bar, 50μm. (C) The protein levels of NPNT and P21 were quantitatively analyzed by Western blot (n=3 per group). (D) The fluorescence results of an EdU assay show the proliferation of HPAEpiC-II cells; Scale bar, 50μm. n=4. Data are presented as mean±SEM. ***P* < 0.01.

**Figure S4**


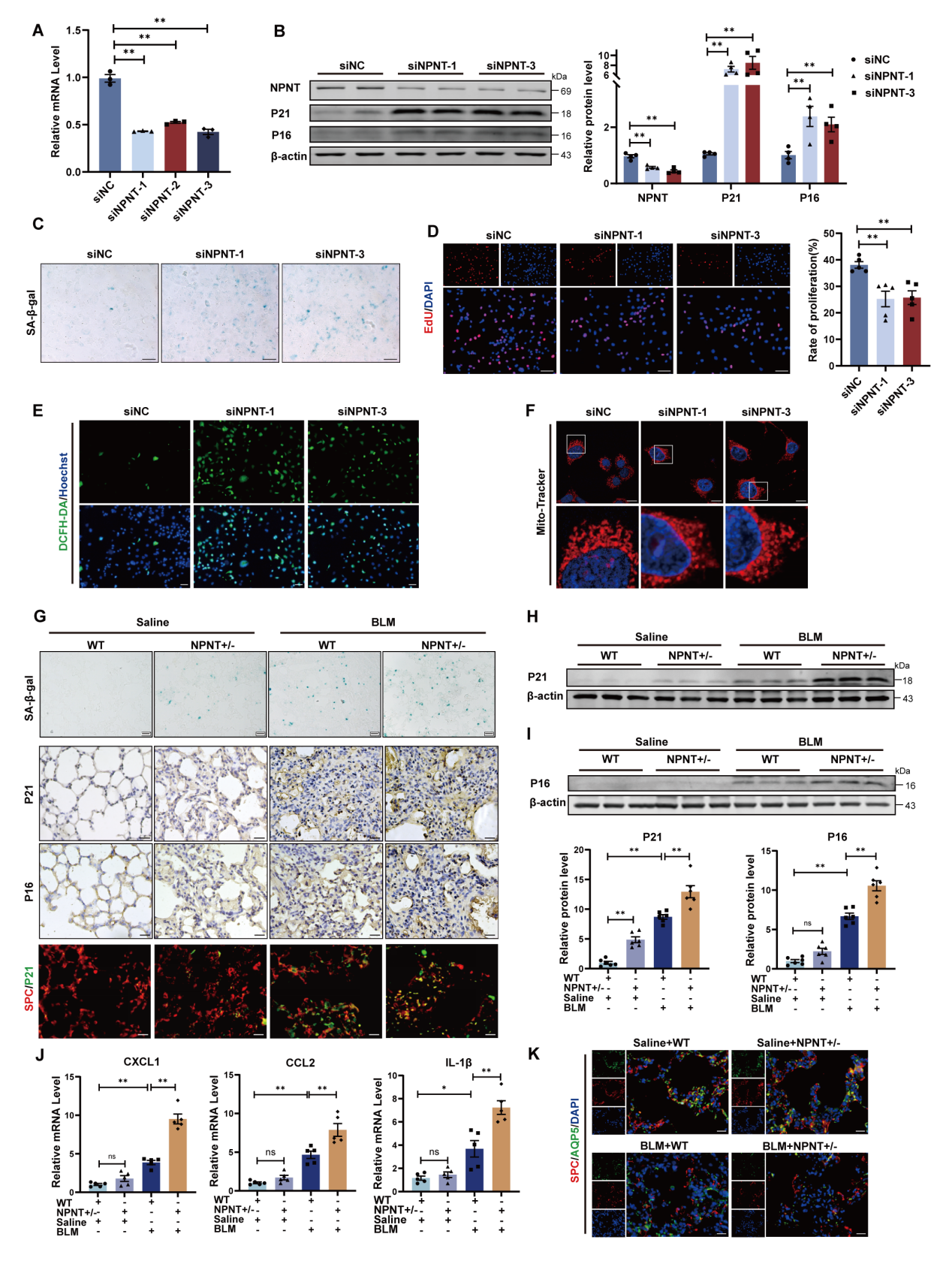


**Supplemental Figure S4**. **The lack of NPNT accelerated the aging process.** (A) Quantitative analysis of silencing efficiency of NPNT siRNA on NPNT mRNA level. n=3 per group. (B) Protein expression of P21 and P16 in MLE-12 cells under NPNT deficient conditions. n=4 per group. (C) Representative images of SA-β-gal staining of cells transfected with siNC or siNPNT. n=5 per group. Scale bar, 50μm. (D) EdU fluorescent staining and quantification showing the the proliferation of MLE-12. n=5 per group. Scale bar, 50μm. (E) Cytosolic ROS level of MLE-12 cells was detected using DCFH-DA probe. n=4 per group. Scale bar, 50μm. (F) Mito-tracker fluorescent staining for mitochondrial morphology. n=4 per group. Scale bar, 15μm. (G) SA-β-gal staining (Scale bar, 50μm) and immunohistochemical staining images of lung tissues of WT and NPNT+/- mice after administration of Saline and BLM. Immunofluorescence staining was used to detect the number of SPC^+^/P21^+^ cells. Scale bar, 20μm. (H-I) Western blotting and quantitative analysis of P21 and P16 protein levels in WT and NPNT+/- mice. n=6 per group. (J) qRT-PCR analysis of CXCL1, CCL2 and IL-1β mRNA levels in lungs of WT and NPNT+/- mice treated with Saline or BLM. n=5 per group. (K) Representative images of frozen lung sections of WT and NPNT+/- mice, with fluorescence display as follows: SPC (Red), AQP5 (Green) and nucleus (Blue). Scale bar, 20μm. Data are presented as mean±SEM. **P* < 0.05, ***P* < 0.01.

**Figure S5**


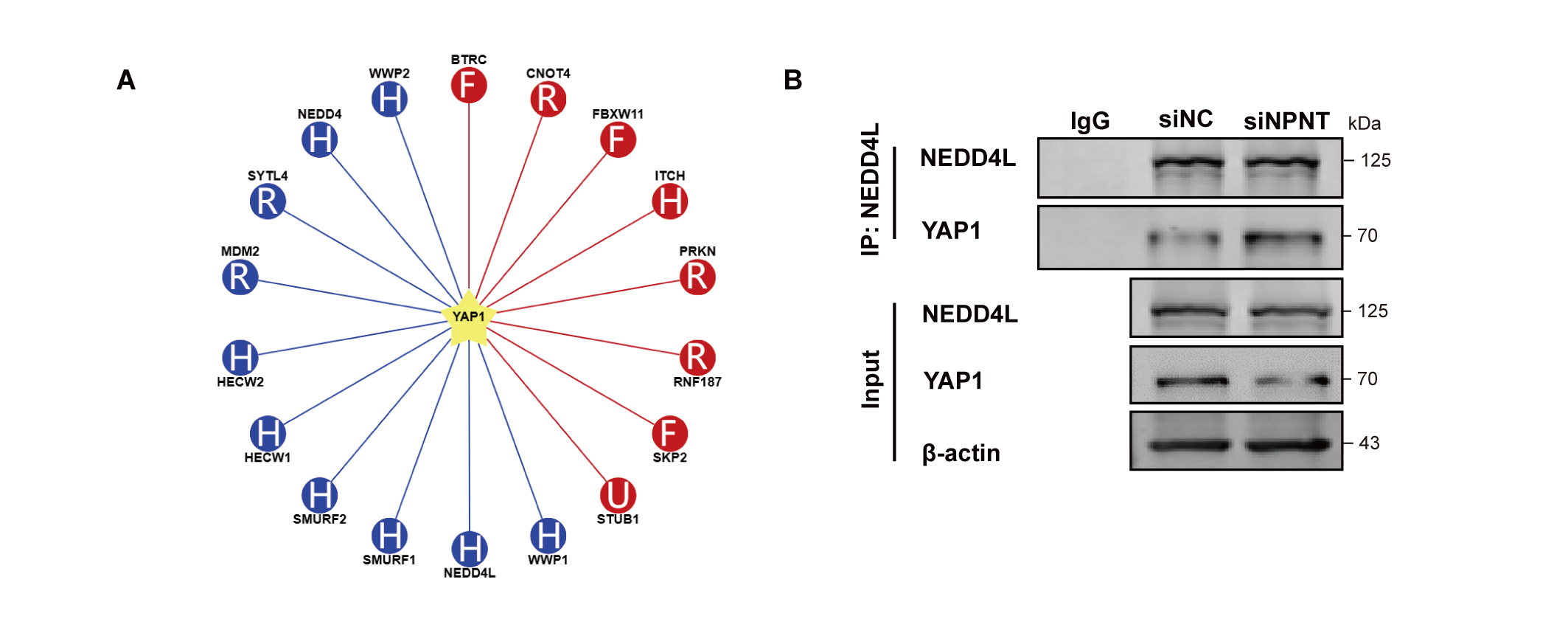


**Supplemental Figure S5. NEDD4L was involved in the ubiquitin-mediated degradation of YAP1-induced by siNPNT.** (A) The E3 ubiquitin ligases acting on YAP1 were predicted with the online database UbiBrowser, an integrated bioinformatics platform. The capital letters in dots indicate the initial letters of E3 ubiquitin ligases-domains: F refers to F-box domain, R refers to RING domain, H refers to HECT domain, U refers to UBOX domain. The predicted interactions are arranged in descending order clockwise based on the confidence score. (B) YAP1 was immunoprecipitated from MLE-12 cell lysates with anti-NEDD4L antibody and detected by Western blot. This confirmed that silencing NPNT regulated the interaction between YAP1 and NEDD4L.

**Figure S6**


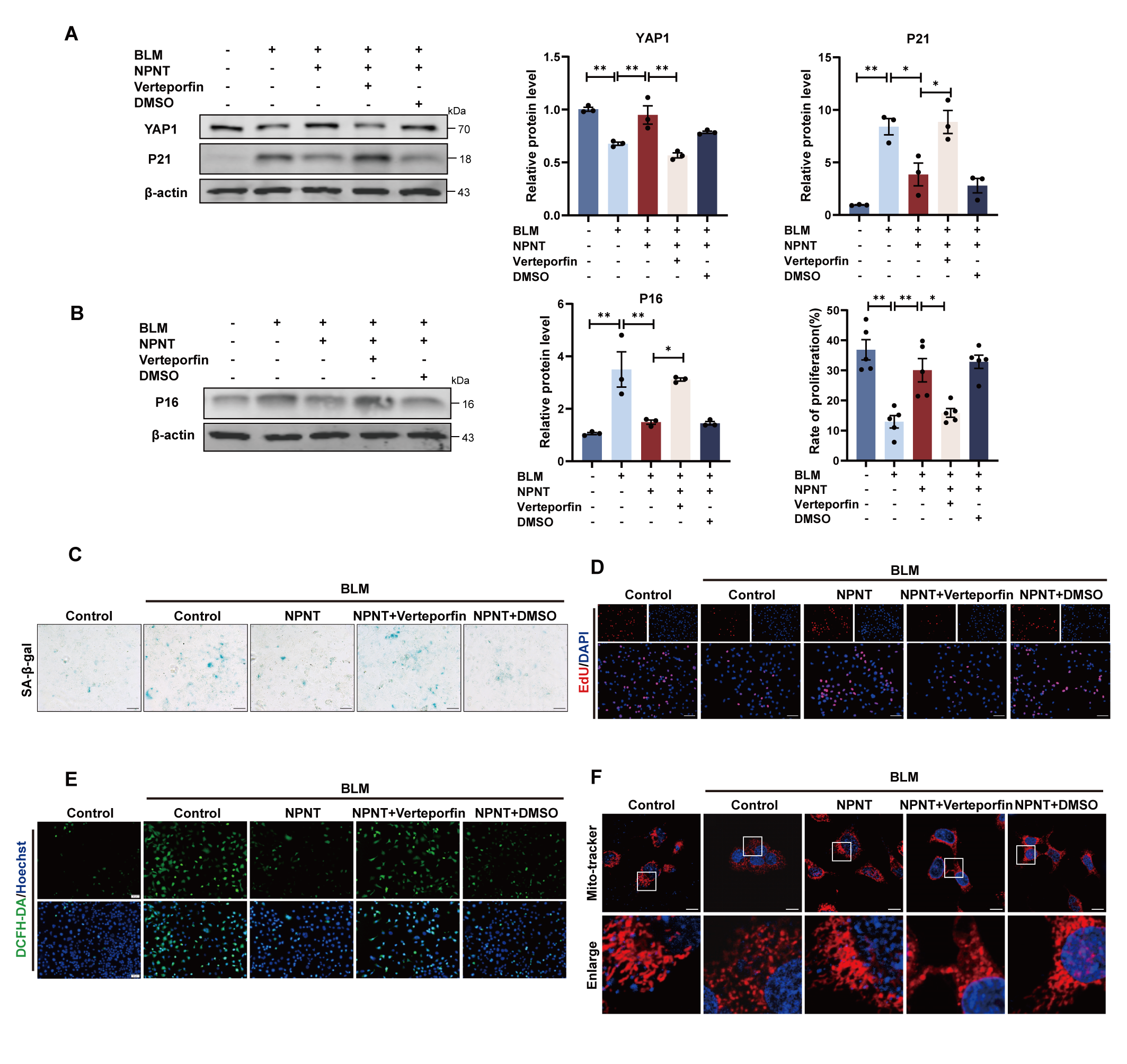


**Supplemental Figure S6.** **YAP1 inhibitor blocked the anti-senescence effect of NPNT.** (A-B) Representative Western blot and statistical data showed the regulatory effect of overexpressing NPNT with or without Verteporfin on P21 and P16 protein levels. n=3 per group. (C) β-galactosidase content in MLE-12 cells after different treatments. n=5 per group. Scale bar, 50μm. (D) The cell proliferation rate was detected by EdU fluorescence staining. Scale bar, 50μm. (E) ROS content in MLE-12 cells treated with or without Verteporfin under the condition of NPNT overexpression. n=5 per group. Scale bar, 50μm. (F) Mito-Tracker staining showed mitochondrial morphology of MLE-12 cells. n=4 per group. Scale bar, 15μm. Data are presented as mean±SEM. **P* < 0.05, ***P* < 0.01.

**Figure S7**


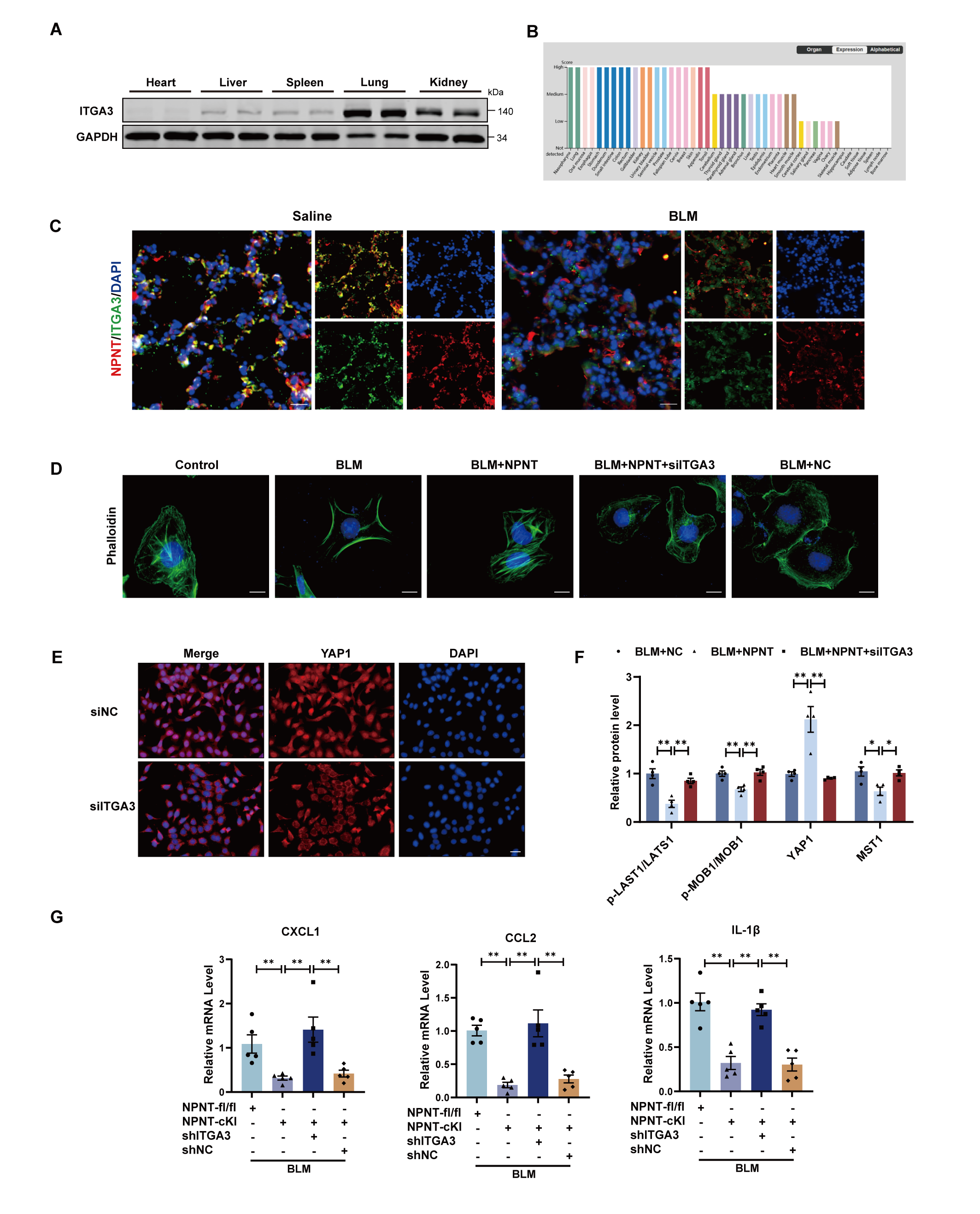


**Supplemental Figure S7**. **ITGA3 colocalized with NPNT and regulated the cytoskeleton.** (A) ITGA3 protein expression bands in different tissues of WT mice. n=4 per group. (B) Protein expression overview of ITGA3 in various tissues. Source of Data: The Human Protein Atlas (https://www.proteinatlas.org/). (C) Fluorescence staining of NPNT and ITGA3 in lung tissue sections of Saline or BLM treated mice. n=4 per group. Scale bar, 20μm. (D) Representative images of phalloidin fluorescence staining in MLE-12 cells with NPNT overexpression and simultaneous knockdown of ITGA3. n=4 per group. Scale bar, 20μm. (E) Immunofluorescence staining was performed to detect the localization of YAP1 in cells transfected with siNC and siITGA3. n=4. Scale bar, 20μm. (F) Quantitative analysis of western blot experimental data. n=3. (G) The mRNA levels of inflammatory factors in each group were measured quantitatively. n=5 per group. Data are presented as mean±SEM. **P* < 0.05, ***P* < 0.01.

**Figure S8**


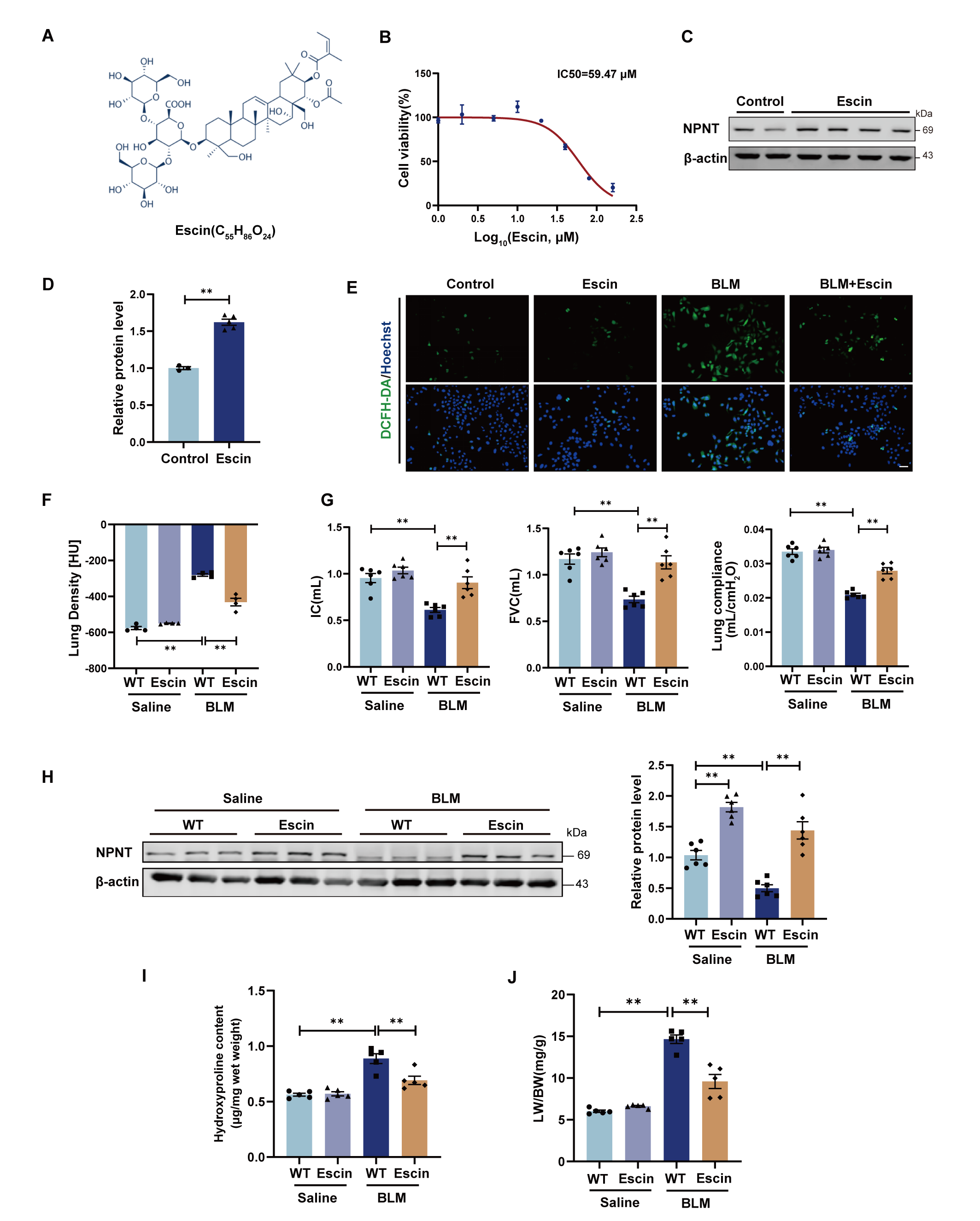


**Supplemental Figure S8**. **Escin enhanced the protein expression of NPNT and ameliorated BLM-induced pulmonary dysfunction in murine models.** (A) The structure of Escin. Molecular formula: C_55_H_86_O_24_. (B) Cell counting kit-8 assay was performed to detect the effect of Escin concentration gradient on cell viability to determine the safe concentration range. n=5 per group. (C-D) Western blotting and quantification showed protein level of NPNT in MLE-12 cells after DMSO or Escin treatment. n=3-5. (E) DCFH-DA fluorescent probe for detecting intracellular ROS content. n=4. Scale bar, 50μm. (F) Quantify lung tissue density based on micro-CT images. n=4. (G) Escin administration restored lung function in BLM-induced mouse model of pulmonary fibrosis. n=6 per group. (H) Western blot and quantification showed the protein levels of NPNT in WT and Escin mice 3 weeks after Saline or BLM injection. n=6 per group. (I) Calculation results of hydroxyproline content. n=5 per group. (J) The ratio of lung weight to body weight in different groups of mice. n=5 per group. Data are presented as mean±SEM. ***P* < 0.01.
